# A next-generation multimetal complex induces immunogenic cell death with distinct transcriptomic signatures

**DOI:** 10.1039/d6cc01066k

**Published:** 2026-04-10

**Authors:** Tomer Babu, Matthew S. Levine, Bingsong Zeng, Jonathan L. Sessler

**Affiliations:** a Department of Chemistry 105 East 24th Street Austin Texas 78712-1224 USA Sessler@cm.utexas.edu

## Abstract

In this study, a Pt(iv)–Au(i)-gemcitabine prodrug that co-releases oxaliplatin, a redox active gold N-heterocyclic carbene (NHC) and gemcitabine upon intracellular reduction is reported. Relative to its constituents, this prodrug displays improved cytotoxicity, metal uptake and an *in vitro* immunogenic cell death response.

## Abstract 2

A next-generation Pt(iv)–Au(i) multimetal complex incorporating gemcitabine enhances cytotoxicity and ICD. RNA-seq pathway analysis reveals transcriptomic signatures predictive of ICD potential (created in BioRender, T. Babu).

## Graphical abstract

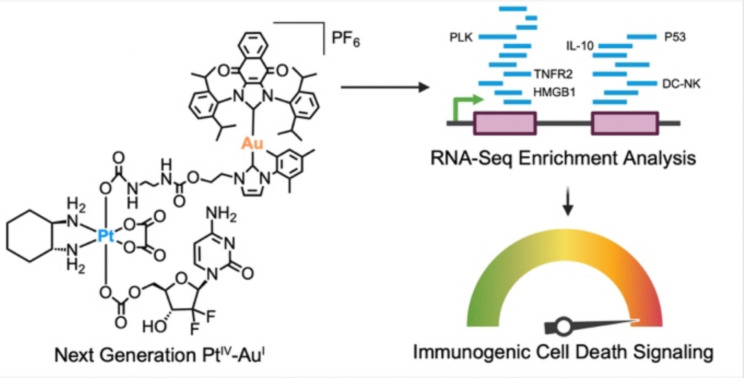

Multimetallic complexes continue to attract interest as platforms capable of integrating distinct modes of biological reactivity within a single metalloconstruct.^1^ By combining multiple metal centers within a well-defined molecular scaffold, it becomes possible to achieve complementary reactivity benefits while avoiding bioavailability and side effect limitations associated with physical drug mixtures.^2^ Here, we demonstrate the promise of this approach *via* a gold N-heterocyclic carbene (NHC) gemcitabine prodrug linked through a platinum center.

Platinum(ii)-based agents, including cisplatin, carboplatin, and oxaliplatin, remain central to chemotherapy, yet severe, long lasting side effects, such as neurotoxicity, ototoxicity and nephrotoxicity, provide limitations to their clinical use.^3^ Most side effects of platinum(ii) agents reflect platinum accumulation in organs that are not associated with the tumor or the tumor microenvironment (TME).^4^ Decreasing the concentration of available platinum at these remote sites could therefore translate into improved clinical outcomes. One possible means of achieving this goal is to use Pt(iv) complexes as prodrugs for the corresponding Pt(ii) species. Pt(iv) complexes are typically hexacoordinate, whereas the FDA-approved Pt(ii) drugs are tetracoordinate. The two additional coordinated moieties, typically referred to as the axial ligands, militate against interactions with various biomolecules, such as histidine- and cysteine-rich proteins.^5^ The additional coordination sites also allow for structural modifications *via* conjugation of ligands that can improve the pharmacological properties of the complex as a whole.^6^ Gibson *et al.* recently reported that Pt(iv) prodrugs have lower metal accumulation in off-target organs, as well as a lower incidence of chemotherapy-induced peripheral neuropathy (CIPN) *in vivo*. CIPN is a long lasting side effect that affects more than 70% of patients receiving Pt(ii) drugs.^7^ Gold(i) complexes have also attracted interest as anticancer agents, in part due to their ability to inhibit members of the thioredoxin reductase (TrxR) family that are often upregulated in malignant cells.^8^ TrxR enzymes are key regulators of cellular redox homeostasis and are found in the cytoplasm (TrxR1) and mitochondria (TrxR2).^9^ Importantly, several gold-based anticancer agents, as well as a number of other metal complexes, have been shown to induce immunogenic cell death (ICD).^10^ ICD is a regulated form of cell death in which tumor cell stress leads to the release of damage-associated molecular patterns (DAMPs), thereby facilitating antigen presentation and activation of an antitumor immune response.^11^ Combining a gold-based TrxR inhibitor^12^ with a Pt(iv) core is attractive as a means to improve the efficacy of both classes of anticancer agents.

We recently reported a first-generation bimetallic complex (Gen 1) that served as a proof-of-concept demonstration of this strategy. Briefly, Gen 1 served to show that co-delivery of both metals can increase an early apoptotic phase, which is important in ICD. However, Gen 1 exhibited modest cytotoxicity and cellular uptake. A rational redesign was therefore implemented to generate a scaffold with improved cytotoxicity that would also induce a robust ICD-associated cellular response.^12^

Herein, the chemical synthesis, characterization, and biological evaluation of a next generation multi-action multimetal complex (Gen 2) is reported (Fig. 1). This new construct incorporates on opposing axial ligand positions both an Au(i) bis-NHC ICD inducer and the FDA approved anticancer drug, gemcitabine. Gemcitabine was chosen for its antiproliferative activity, relatively high water solubility, and ability to potentiate an immune response^13^ as well as the fact that it is co-administered with oxaliplatin in the clinic.^14^ As detailed below, Gen 2 demonstrates improved cytotoxicity compared to Gen 1 and various monometallic controls. Additionally, Gen 2 displayed good nuclear and mitochondrial co-accumulation. It also induced recognized ICD-related DAMPs, including (i) calreticulin (CRT) translocation to the cell membrane, (ii) transcriptional activation of high mobility group box 1 protein (HMGB1), (iii) tumor necrosis factor 2 (TNFR2), (iv) p53, (v) dendritic cell and natural killer cell (DC–NK) signaling, and (vi) G_2_/M checkpoint pathways. The transcriptional effects observed for Gen 2 are distinct from those triggered by oxaliplatin and gemcitabine, highlighting the unique multi-action behavior arising from covalent incorporation of multiple agents within a single prodrug.

**Fig. 1:**
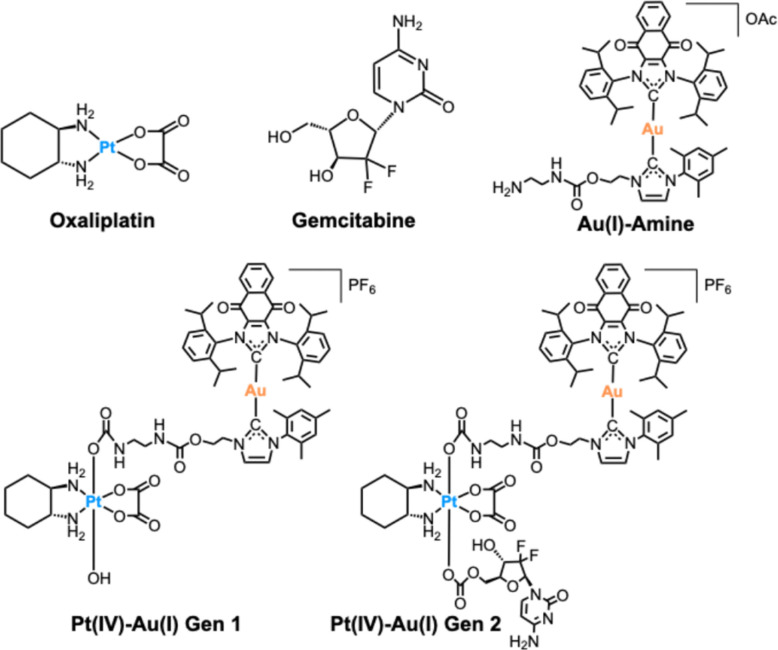
Chemical structures of the compounds used in this study.

The synthetic route to Pt(iv)–Au(i) Gen 2 is shown in Scheme S1 (SI). Briefly, an asymmetric Au(i) bis NHC was prepared by reacting chloro(dimethylsulfide)gold(i) with a redox active 1,4-naphthoquinone NHC to give the corresponding NHC–Au–Cl complex in high yield. The chlorido ligand was first replaced by hydroxido and subsequently by an acetonyl ligand. The resulting acetonyl derivative enabled the preparation of the bis-Au(i)–NHC complex in high yield.^15^ In order to facilitate the conjugation to Pt(iv), this hydroxyl was reacted with 1,1′-carbonyldiimidazole and subsequently with ethylenediamine yielding a primary amine end group. Separately, oxaliplatin(iv) bearing two hydroxyls was conjugated first to gemcitabine bearing Boc protecting groups on the amine and 3′-OH sites. Next, the protecting groups were removed by treating with a nitric acid:CH_2_Cl_2_ mixture for 10 min. TFA was avoided in this step as it can coordinate to the *trans* axial position.^16^ This gemcitabine-containing intermediate was then reacted with disuccinimidyl carbonate and subsequently with the primary amine of the bis NHC Au(i) using a carbamate strategy^17^ to yield the multimetal complex in 33% yield following purification using preparative HPLC.

The Gen 2 prodrug was characterized by ^1^H, ^13^C, and ^195^Pt spectroscopies, as well as by HRMS (Fig. S16–S20, SI). Its physicochemical properties, including stability (*t*_1/2_) under physiological conditions and propensity to undergo reduction, were then assessed (Fig. S21 and S22, SI). A key criterion for a prodrug is that it remains largely intact until it reaches its target.^18^ Stability was assessed by dissolving the complex in 1% DMSO in RPMI 1640 cell culture medium at 37 °C and monitoring changes (if any) by HPLC. The ability of Gen 2 to undergo reduction in the presence of a 10-fold excess of ascorbate (two electron donor), GSH (one electron donor) or a mixture of both to simulate biological redox conditions at pH 7.0, 37 °C was also assessed by HPLC. It was found to be reduced rapidly under these conditions, leading to the concurrent release of the Au(i) complex, the gemcitabine axial ligand, and free oxaliplatin as confirmed by LC-MS (Fig. S21, SI). On the other hand, in the absence of a reductant, Gen 2 proved stable under physiological conditions with a *t*_1/2_ for decomposition of 51 h (Fig. S22, SI).

Cytotoxicity was then assessed in CT26 colorectal carcinoma cells using a 3-(4,5-dimethylthiazol-2-yl)-2,5-diphenyltetrazolium bromide (MTT) assay. Both Gen 2 and gemcitabine displayed steep dose–response curves and sub-nanomolar IC_50_ values that were lower than those observed for Gen 1 and its monometallic precursors (Fig. 2). A similar trend was also found in A549 human lung carcinoma cells where Gen 2 produced low nanomolar IC_50_ values, comparable to gemcitabine and approximately two orders of magnitude lower than oxaliplatin or Gen 1 (Table S1, SI). On this basis, we suggest that cancer cell death is mediated primarily through the gemcitabine, which is released from the prodrug inside the cancer cells.

**Fig. 2:**
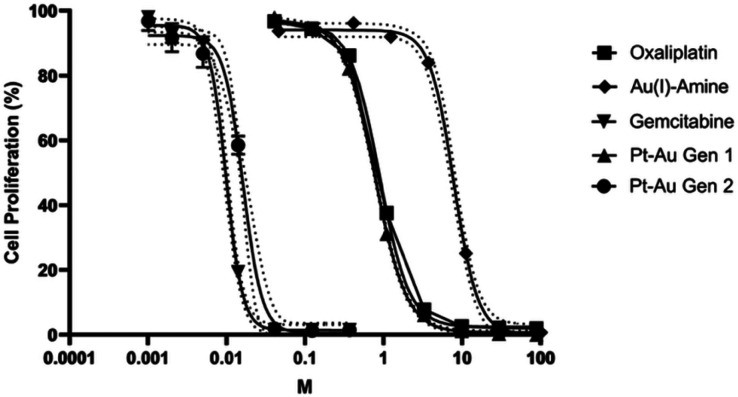
Dose–response proliferation of CT26 cells seen upon incubation with monometallic and multimetallic complexes (*t* = 72 h). Data represent the mean ± SD from 3 independent experiments.

To determine whether the enhanced antiproliferative activity of Gen 2 is further driven by altered intracellular uptake, the total cellular uptake of platinum and gold was quantified by inductively coupled plasma mass spectrometry (ICP-MS). CT26 cells were treated for 24 h with equimolar concentrations of oxaliplatin, Au(i)–amine, Pt–Au (Gen 1), and Pt–Au (Gen 2) (Fig. 3, top). Gen 2 exhibited a statistically significant increase in total platinum uptake relative to both oxaliplatin and Gen 1. In contrast, gold uptake was reduced compared to what was seen for Au(i)–amine; however, it proved significantly higher than that observed for Gen 1. These findings were taken as evidence of improved co-delivery of both metals (platinum and gold) by the second-generation Gen 2 scaffold.

**Fig. 3:**
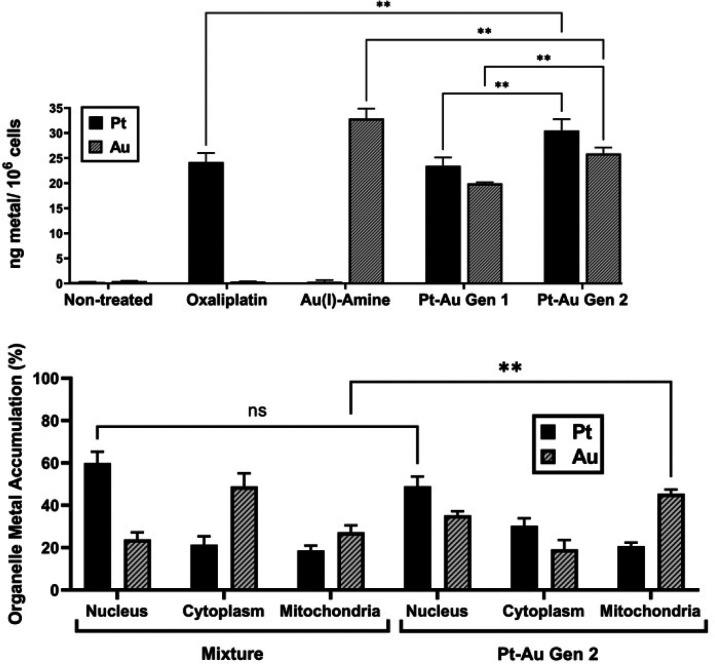
(Top) Total cellular metal uptake of various complexes. (Bottom) Organelle metal accumulation of a 1 : 1 : 1 mixture of oxaliplatin : Au(i)–amine : gemcitabine *versus* Gen 2. **p < 0.01 (two-way ANOVA).

To further probe the subcellular metal distribution following intracellular reduction, nuclei, cytoplasm, and mitochondria were isolated from CT26 cells incubated for 24 h with equimolar concentrations of either (1) a 1 : 1 : 1 physical mixture of oxaliplatin, Au(i)–amine, and gemcitabine, or (2) Gen 2 (Fig. 3, bottom). In both cases, platinum predominantly localized to the nucleus. No statistically significant difference in nuclear platinum levels was observed between the two treatments. In contrast, Gen 2 displayed higher gold content within the mitochondrial fraction relative to the physical mixture.

Based on the above, we suggest that incorporation of platinum, gold and a nucleoside drug, gemcitabine, within a single prodrug increases the total cellular accumulation of both metals compared to Gen 1 and modifies the intracellular distribution relative to a physical mixture of the constituents. The enhanced cytotoxicity of Gen 2 is thus not solely attributable to axial gemcitabine release but is accompanied by increased platinum uptake and altered subcellular distribution of the metal components following reduction. Such changes in metal uptake and localization may lead to a diverse cellular response. This prompted us to investigate the ICD potential of Gen 2 *in vitro*.

Transcriptional analysis of ICD has largely focused on immune cell populations or TME remodeling.^19^ In contrast, pathway-level transcriptional responses within tumor cells after chemotherapeutic treatment, and their relevance to ICD-associated signaling, are underexplored.^20^ We therefore sought to determine whether Gen 2 elicits canonical gene expression pathways that are linked to ICD. RNA profiling was performed on RNA isolated from CT26 cells treated with either Gen 2, oxaliplatin, or gemcitabine. Oxaliplatin and gemcitabine are FDA-approved drugs with well-established mechanisms of action (MOAs). They were therefore used as controls. The differential expression for the three groups was analyzed by ingenuity pathway analysis (IPA) and compared to untreated cells (Fig. S25–S27, SI). This approach evaluates changes across groups of functionally related genes rather than isolated transcriptional events, allowing in principle a broader understanding of downstream cellular response impact.^21^

The pathways affected by oxaliplatin were related to upregulation of oxidative phosphorylation, Eukaryotic Translation Initiation Factor 2 (EIF2) signaling and inhibition of mitochondrial dysfunction (Fig. S24, SI). This correlates with the findings of Sastre-Serra *et al.* that led to oxidative phosphorylation being suggested as a predictive biomarker for oxaliplatin response in colorectal cancer.^22^ In the case of gemcitabine, the pathways affected were associated with pro-inflammatory responses (Fig. S25, SI), a finding consistent with the immunomodulatory functions of gemcitabine in cancer.^23^ In contrast, Gen 2 was found to upregulate signaling pathways associated with an immunogenic response (Fig. 4). Activation of G2/M DNA damage checkpoint signaling was observed. This leads us to suggest that Gen 2 causes cell cycle arrest and death to occur at the G2/M transition phase. Polo-like kinase (PLK) signaling pathway inhibition was seen and provides further evidence in support of the hypothesis that Gen 2 causes cell cycle arrest and death at the G2/M transition phase as these kinases are essential for cell cycle regulation beginning at the G2/M phase.^24^

**Fig. 4:**
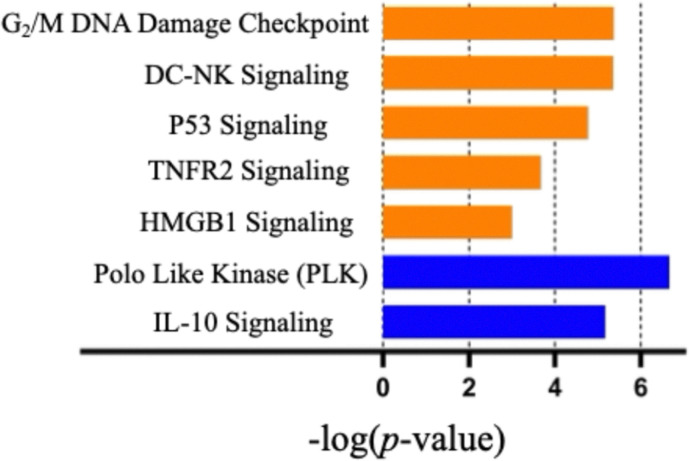
Ingenuity pathway analysis of selected canonical signaling pathways of Gen 2 vs. untreated CT26 cells. Color is based on *Z*-score with orange and blue colors indicating upregulated and downregulated pathways, respectively (for the associated gene list, see Fig. S28, SI).

The activation of p53 signaling is a cellular response to stress, such as oxidation and DNA damage, that is associated with oxaliplatin and Au(i) naphthoquinone NHC.^25,26^ The activation of HMGB1 signaling and TNFR2 signaling is consistent with Gen 2 causing inflammation due to activation of inflammatory cells through HMGB1 and other activating proteins correlated with induction of an immune response.^27,28^ Gen 2-induced inflammatory signaling coincides with the upregulated crosstalk between DC-NK signaling that is required for the activation and subsequent immune cell amplification associated with the activation of both an innate and adaptive immune response.^29^ Inhibition of the interlukin-10 (IL-10) signaling pathway was also seen. The IL-10 signaling pathway is mostly associated with an anti-inflammatory response leading us to propose that inflammation effects caused by Gen 2 are enhanced. Additionally, the cytokine IL-10 has been shown to downregulate T-cell function and thus help tumors evade detection by the immune system.^30^ It is reasonable to expect that inhibition of this pathway would result in low levels of IL-10 within the TME and hence an active immune response.^31^ Taken in concert, the concurrent activation/inhibition of these pathways is associated with dying cancer cells that promote inflammatory signaling and activation of both innate and adaptive immunogenic responses. In order to validate ICD potential from RNA sequencing, we compared Gen 2 ATP and HMGB1 release from CT26 cells. The total release of both DAMPs was significantly higher compared to oxaliplatin and gemcitabine (S23 and S24, SI).

During ICD, CRT translocates from the endoplasmic reticulum (ER) to the cell surface of tumor cells and acts as a pro-phagocytic “eat me” signal for antigen presenting cells (APCs).^32^ As translocation is a post-translational process and cannot be inferred from RNA sequencing data, we examined CRT translocation to evaluate ICD induction at the cellular level. Confocal microscopy of CT26 cells treated with either oxaliplatin, gemcitabine, or Gen 2 exhibited increased co-localization of emission signals from a commercially available CRT antibody (green) with cell membrane immunostaining with wheat-germ agglutinin (WGA) antibody (red). Gen 2 exhibited the greatest amount of CRT translocation compared to oxaliplatin and gemcitabine (Fig. 5). The findings thus provide evidence that the immunogenic response elicited by the multimetal complex, Gen 2, is enhanced relative to its constituent agents.

**Fig. 5:**
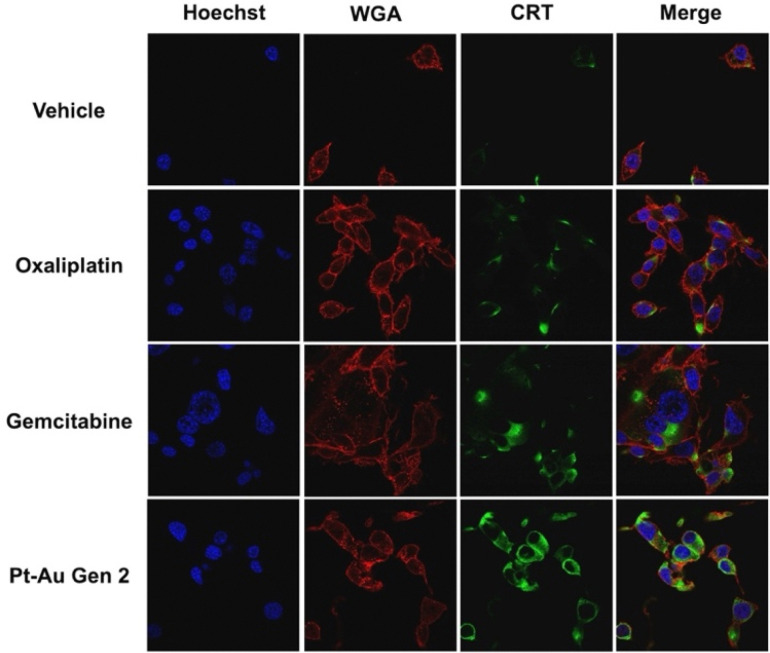
Confocal microscopy studies of CRT translocation after 4 h of incubation.

In summary, we report a next-generation Pt(iv)–Au(i) multimetal prodrug that integrates platinum-based DNA damage, gold-mediated redox stress, and nucleoside-driven antiproliferative activity within a single, reduction-activated scaffold. We believe the approach described here may have a role to play in the development of next generation drugs.

## Conflicts of interest

There are no conflicts to declare.

## Supplementary Material

CC-062-D6CC01066K-s001

## Data Availability

Data supporting this publication can be obtained from the supplementary information (SI). See DOI: https://doi.org/10.1039/d6cc01066k.
