# Supplementary material for: A next-generation multimetal complex induces immunogenic cell death with distinct transcriptomic signatures
Source: Chem Commun (Camb). 2026 Apr 10;62(35):8952–5. doi: 10.1039/d6cc01066k (PMC13093250; doi:10.1039/d6cc01066k)
Supplement: CC-062-D6CC01066K-s001 [file CC-062-D6CC01066K-s001.pdf]

Supplementary Information (SI) for ChemComm.
This journal is © The Royal Society of Chemistry 2026

Next-generation multimetal complex induces immunogenic cell death with distinct transcriptomic signatures

Tomer Babu[a], Matthew S. Levine[a], Bingsong Zeng[a] and Jonathan L. Sessler*[a]

[a] Department of Chemistry
The University of Texas at Austin
105 East 24th Street, Austin, Texas 78712-1224, United States

Corresponding Author Email - Sessler@cm.utexas.edu

Table of Contents

1. Experimental Section 5

1.1 Material and Methods 5

1.2. Synthesis 5

(1,3-bis(2,6-Diisopropylphenyl)-4,9-dioxo-1,3,4,9-tetrahydro-2H-2λ3-naphtho[2,3-d]imidazol-2-yl)(1-(2-hydroxyethyl)-3-mesityl-1,3-dihydro-2H-2λ3-imidazol-2-yl) gold(I) (complex 1) 5

(1-(2-((1H-Imidazole-1-carbonyl)oxy)ethyl)-3-mesityl-1,3-dihydro-2H-2λ3-imidazol-2-yl)(1,3-bis(2,6-diisopropylphenyl)-4,9-dioxo-1,3,4,9-tetrahydro-2H-2λ3-naphtho[2,3-d]imidazol-2-yl) gold(I) 6

(1-(2-(((2-Aminoethyl)carbamoyl)oxy)ethyl)-3-mesityl-1,3-dihydro-2H-2λ3-imidazol-2-yl)(1,3-bis(2,6-diisopropylphenyl)-4,9-dioxo-1,3,4,9-tetrahydro-2H-2λ3-naphtho[2,3-d]imidazol-2-yl)gold (I) Acetate salt (3) 6

$Oxali^{IV}$(Gem)(Au(I)) – Gen 1 7

cct-[Pt((1R,2R)-DACH)(ox)(Gem-Bis BOC protected)(OH)] (5) 7

cct-[Pt((1R,2R)-DACH)(ox)(gemcitabine)(OH)] (Oxali(Gem)(OH) (6) 8

cct-[Pt((1R,2R)-DACH)(ox)(gemcitabine)(succinimidyl carbonate)] (7) 8

$Oxali^{IV}$(Gem)(Au(I)) – Gen 2 8

1.3. Reduction and Stability 9

1.4. Antiproliferative assay in tumorigenic cells 9

1.5. Total Cellular Metal Accumulation 10

1.6. Metal Accumulation in Organelles 10

1.7. Extracellular ATP release 10

1.8. HMGB1 release 10

1.9. CRT Translocation Confocal Microscopy 11

2.0. RNA Sequencing (RNA-Seq) 11

Scheme 1 Synthetic route to Oxali(Gem)(Au(I)) – Gen 2 13

Figure S1 ^{1}H NMR spectrum of (1,3-bis(2,6-diisopropylphenyl)-4,9-dioxo-1,3,4,9-tetrahydro-2H-2λ3-naphtho[2,3-d]imidazol-2-yl)(1-(2-hydroxyethyl)-3-mesityl-1,3-dihydro-2H-2λ3-imidazol-2-yl) gold(I) in DMSO-d_6 13

Figure S2 HRMS (1,3-bis(2,6-diisopropylphenyl)-4,9-dioxo-1,3,4,9-tetrahydro-2H-2λ3-naphtho[2,3-d]imidazol-2-yl)(1-(2-hydroxyethyl)-3-mesityl-1,3-dihydro-2H-2λ3-imidazol-2-yl) gold(I) 14

Figure S3 HPLC chromatogram of (1,3-bis(2,6-diisopropylphenyl)-4,9-dioxo-1,3,4,9-tetrahydro-2H-2λ3-naphtho[2,3-d]imidazol-2-yl)(1-(2-hydroxyethyl)-3-mesityl-1,3-dihydro-2H-2λ3-imidazol-2-yl) gold(I) - 0-100% acetonitrile in 5.84 min + 2 min constant 100% acetonitrile.... 14

Figure S4 ^{1}H NMR spectrum of (1-(2-((1H-imidazole-1-carbonyl)oxy)ethyl)-3-mesityl-1,3-dihydro-2H-2λ3-imidazol-2-yl)(1,3-bis(2,6-diisopropylphenyl)-4,9-dioxo-1,3,4,9-tetrahydro-2H-2λ3-naphtho[2,3-d]imidazol-2-yl) gold(I) in DMSO-d_6 15

Figure S5 ^{13}C NMR spectrum of (1-(2-((1H-imidazole-1-carbonyl)oxy)ethyl)-3-mesityl-1,3-dihydro-2H-2λ3-imidazol-2-yl)(1,3-bis(2,6-diisopropylphenyl)-4,9-dioxo-1,3,4,9-tetrahydro-2H-2λ3-naphtho[2,3-d]imidazol-2-yl) gold(I) in DMSO-d_6 16

Figure S6 HRMS of (1-(2-((1H-imidazole-1-carbonyl)oxy)ethyl)-3-mesityl-1,3-dihydro-2H-2λ3-imidazol-2-yl)(1,3-bis(2,6-diisopropylphenyl)-4,9-dioxo-1,3,4,9-tetrahydro-2H-2λ3-naphtho[2,3-d]imidazol-2-yl) gold(I) 16

Figure S7 ^{1}H NMR spectrum of (1-(2-(((2-Aminoethyl)carbamoyl)oxy)ethyl)-3-mesityl-1,3-dihydro-2H-2λ3-imidazol-2-yl)(1,3-bis(2,6-diisopropylphenyl)-4,9-dioxo-1,3,4,9-tetrahydro-2H-2λ3-naphtho[2,3-d]imidazol-2-yl)gold (I) in DMSO-d_6 17

Figure S8 ^{13}C NMR spectrum of (1-(2-(((2-Aminoethyl)carbamoyl)oxy)ethyl)-3-mesityl-1,3-dihydro-2H-2λ3-imidazol-2-yl)(1,3-bis(2,6-diisopropylphenyl)-4,9-dioxo-1,3,4,9-tetrahydro-2H-2λ3-naphtho[2,3-d]imidazol-2-yl)gold (I) in DMSO-d_6 17

Figure S9 HPLC chromatogram of (1-(2-(((2-Aminoethyl)carbamoyl)oxy)ethyl)-3-mesityl-1,3-dihydro-2H-2λ3-imidazol-2-yl)(1,3-bis(2,6-diisopropylphenyl)-4,9-dioxo-1,3,4,9-tetrahydro-2H-2λ3-naphtho[2,3-d]imidazol-2-yl)gold (I) - 0-100% acetonitrile in 5.84 min + 2 min constant 100% acetonitrile.......... 18

Figure S10 ESI-HRMS (+ve) of (1-(2-(((2-Aminoethyl)carbamoyl)oxy)ethyl)-3-mesityl-1,3-dihydro-2H-2λ3-imidazol-2-yl)(1,3-bis(2,6-diisopropylphenyl)-4,9-dioxo-1,3,4,9-tetrahydro-2H-2λ3-naphtho[2,3-d]imidazol-2-yl)gold (I) 18

Figure S11 HPLC chromatogram of Oxali(Gem-Bis BOC protected)(OH)- 0-100% acetonitrile in 5.84 min + 2 min constant 100% acetonitrile. 19

Figure S12 ^{195}Pt NMR spectrum of Oxali(Gem-Bis BOC protected)(OH) 19

Figure S13 HPLC chromatogram of Oxali(Gem)(OH)- 0-100% acetonitrile in 5.84 min + 2 min constant 100% acetonitrile. 20

Figure S14 HPLC chromatogram of Oxali(Gem)(MSC)- 0-100% acetonitrile in 5.84 min + 2 min constant 100% acetonitrile. 20

Figure S15 ^{195}Pt NMR spectrum of Oxali(Gem)(MSC) 21

Figure S16 ^{1}H NMR spectrum of Oxali(Gem)(Au(I)) – Gen 2 in DMSO-d_6 21

Figure S17 ^{13}C NMR spectrum of Oxali(Gem)(Au(I)) – Gen 2 in DMSO-d_6 22

Figure S18 HPLC chromatogram of Oxali(Gem)(Au(I)) – Gen 2 - 0-100% acetonitrile in 5.84 min + 2 min constant 100% acetonitrile. 22

Figure S19 ^{195}Pt NMR spectrum of Oxali(Gem)(Au(I)) – Gen 2 23

Figure S20 HRMS of Oxali(Gem)(Au(I)) – Gen 2 23

Figure S21 (A) Pt(IV)-Au(I) Gen 2 reduction products – Reduction of prodrug incubated for 75 min with 10 equiv. Asc. at pH 7 and 37 ºC as monitored via LC-MS. (B) Half-life of Pt(IV)-Au(I) Gen 2 in presence of excess GSH. (C) Half-life of Pt(IV)-Au(I) Gen 2 in presence of excess GSH and ascorbic acid. 24

Table S1 - IC_{50} values [μM] in A549 after a 72 h incubation period (tested in triplicate). 25

Figure S22 Stability study of Pt(IV)-Au(I) Gen 2 – A) HPLC chromatogram over time. B) Linear fitting of $\ln(A_t/A_0)$ vs time (t) for $t_{1/2}$ calculation. 25

Figure S23 Extracelluar ATP release 26

Figure S24 HMGB1 release 26

Figure S25 RNA pathway analysis of Oxali(Gem)(Au(I)) – Gen 2 versus untreated CT26 cells 27

Figure S26 RNA pathway analysis of oxaliplatin versus untreated CT26 cells 28

Figure S27 RNA Pathway Analysis of Gemcitabine versus untreated CT26 cells 29

Figure S28 RNA Microarray Analysis –Associate Genes with Canonical Pathways 30

References 31

1. Experimental Section

1.1 Material and Methods

All chemical reactions were conducted under nitrogen atmosphere using Schlenk techniques unless otherwise noted. The glassware was oven dried at 120°C before use. All materials were obtained from commercial sources at the highest purity available and used without further purification. Chemicals were purchased from Sigma-Aldrich. Solvents were either dried using a solvent purification system (dichloromethane (DCM), acetonitrile, methanol) or dried over molecular sieves (toluene) (3 Å) and degassed prior to use. Complexes were characterized by ^{1}H & ^{195}Pt NMR spectroscopy, HRMS, and elemental analyses. Progress of reactions was monitored by analytical HPLC (Thermo Scientific UltiMate 3000) with a reverse-phase C18 column Phenomenex Kinetex, length 100 mm, internal diameter 4.60 mm, particle size 2.6 µm, pore size 100 Å. The purity and retention time (RT) of the newly synthesized complex (Gen 2) was measured with the same analytical HPLC system water/acetonitrile gradient at a flow rate of 1 mL/min. Reaction mixtures were purified on a preparative HPLC system (Thermo Scientific UltimaMate 3000 station) equipped with a reverse-phase C18 column (Phenomenex Luna 250 × 21.2 mm, 10 µm, 100 Å) using a similar mobile phase at a flow rate of 15 mL/min. UV spectroscopic detection was measured at 220 nm in the case of both the HPLC systems. Appropriate fractions were combined and lyophilized to yield the desired pure compounds.

All NMR spectroscopic data were collected on a Bruker AVANCE III^{TM} HD 500 MHz spectrometer or a Varian Inova 400 MHz. The data were processed using either MestreNova software. ^{1}H NMR chemical shifts were referenced with the individual solvent residual peaks of the respective NMR solvents. ^{195}Pt NMR chemical shifts were reported with respect to the chemical shift of a standard (K_2PtCl_4 in water) at −1624 ppm. High resolution mass spectra (HR-MS) were recorded using a X500R QTOF by direct injection $^{+}$ve mode electrospray ionization.

1.2. Synthesis

(1,3-bis(2,6-Diisopropylphenyl)-4,9-dioxo-1,3,4,9-tetrahydro-2H-2λ3-naphtho[2,3-d]imidazol-2-yl)(1-(2-hydroxyethyl)-3-mesityl-1,3-dihydro-2H-2λ3-imidazol-2-yl) gold(I) (complex **1**)

This Au(I) complex was prepared using a reported procedure.[1]

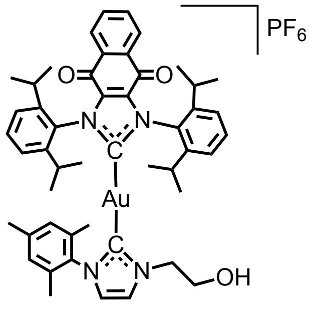

1

^{1}H NMR (500 MHz, DMSO-d_6) δ 8.03 (dd, J = 5.7, 3.4 Hz, 2H), 7.91 (dd, J = 5.9, 3.3 Hz, 2H), 7.63 (t, J = 7.8 Hz, 2H), 7.51 (d, J = 1.8 Hz, 1H), 7.34 (d, J = 7.8 Hz, 4H), 7.28 (d, J = 1.8 Hz, 1H), 6.82 (s, 2H), 4.93 (t, J = 4.9 Hz, 1H), 3.58 (t, J = 5.0 Hz, 2H), 3.23 (q, J = 5.0 Hz, 2H), 2.58 – 2.51 (m, 8H), 2.40 (s, 3H), 1.56 (s, 6H), 1.05 (dd, J = 6.8, 2.8 Hz, 20H). HPLC – 6.7 min (0-100% ACN in 5.84 min + 2 min constant 100% ACN), ESI-HRMS (m/z) calculated for $[C_{49}H_{56}AuN_4O_3]^{+}$: 945.98, found 945.39.

(1-(2-((1H-Imidazole-1-carbonyl)oxy)ethyl)-3-mesityl-1,3-dihydro-2H-2λ3-imidazol-2-yl)(1,3-bis(2,6-diisopropylphenyl)-4,9-dioxo-1,3,4,9-tetrahydro-2H-2λ3-naphtho[2,3-d]imidazol-2-yl) gol d(I)

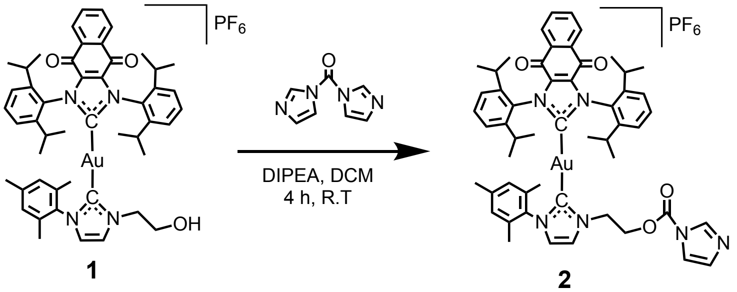

To an oven-dried 25-mL three-neck round bottom flask was added 1,1'-carbonyldiimidazole (187 mg, 1.15 mmol, 6 equiv.). After purging with N_2, 2 mL dry DCM and diisopropylamine (DIPEA) (50.3 μL, 0.289 mmol, 1.5 equiv.) were added to the flask. A solution of compound [**1**][PF_6] (211 mg, 0.192 mmol, 1 equiv.) in 4 mL dry DCM was made up in an oven-dried 10-mL scintillation vial and added dropwise to the flask over the course of 10 min. The mixture was stirred at room temperature for 4 h to produce a clear yellow liquid. The mixture was concentrated under reduced pressure and purified on silica gel with 10% MeOH/DCM as the mobile phase. The fractions containing pure product were collected and dried *in vacuo* to afford **2** as a yellow solid. Yield: 184 mg (80.3%) ESI-HRMS (acetonitrile) (m/z): calculated for $C_{53}H_{58}AuN_6O_4$: 1039.4180, observed: 1039.4171. ^{1}H NMR (600 MHz, DMSO-d_6) δ 8.08 (s, 1H), 8.01 (dd, J = 5.7, 3.3 Hz, 2H), 7.89 (dd, J = 5.8, 3.3 Hz, 2H), 7.81 (d, J = 2.0 Hz, 1H), 7.61 (t, J = 7.8 Hz, 2H), 7.42 (s, 1H), 7.32 (m, 5H), 7.05 (s, 1H), 6.80 (s, 2H), 4.26 (t, J = 4.9 Hz, 2H), 3.93 (t, J = 5.0 Hz, 2H), 2.52 (m, 4H), 2.38 (s, 3H), 1.51 (s, 6H), 1.02 (dd, J = 6.8, 4.8 Hz, 24H), ^{13}C NMR (151 MHz, dmso) δ 192.72, 181.06, 174.97, 148.03, 145.25, 138.99, 137.52, 135.06, 134.13, 133.95, 133.43, 133.06, 132.48, 131.26, 130.94, 129.69, 126.72, 124.46, 124.44, 123.84, 117.79, 66.38, 65.36, 49.06, 40.39, 40.25, 40.11, 39.97, 39.83, 39.69, 39.56, 28.35, 24.55, 23.97, 21.24, 17.02, 15.62.

(1-(2-(((2-Aminoethyl)carbamoyl)oxy)ethyl)-3-mesityl-1,3-dihydro-2H-2λ3-imidazol-2-yl)(1,3-bis(2,6-diisopropylphenyl)-4,9-dioxo-1,3,4,9-tetrahydro-2H-2λ3-naphtho[2,3-d]imidazol-2-yl)gold (I) Acetate salt (**3**)

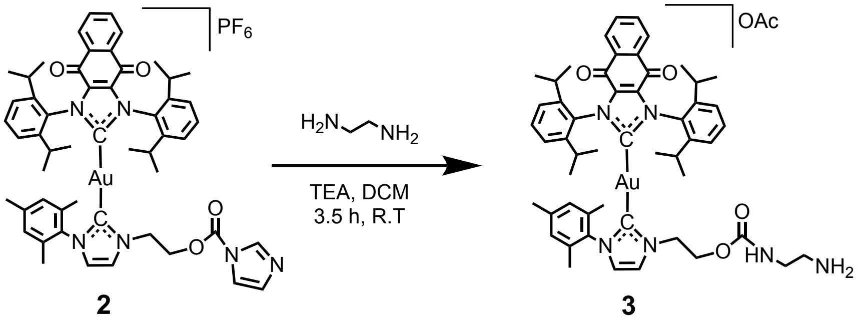

To an oven-dried 10-mL two-neck round bottom flask was added compound **2** (68.0 mg, 57.4 μmol, 1 equiv.). After purging with N_2, 4 mL dry DCM was added to the flask. Ethylenediamine (23.0 μL, 344 μmol, 6 equiv.) and TEA (12.0 μL, 86.1 μmol, 1.5 equiv.) were then added. The mixture was stirred at room temperature for 3.5 h to produce a yellow clear liquid. The mixture was subject to flash column chromatography (silica gel, DCM : MeOH (with 7% w/v NH_4OAc) =

98:2 and then 80:20). The volatiles in the fractions were evaporated under reduced pressure, and the remaining aqueous suspension was lyophilized for 48 h to afford a light yellow solid. Yield: 62.4 mg (92%). ESI-HRMS (acetonitrile) (m/z): calculated for $C_{52}H_{62}AuN_6O_4$: 1031.4493, observed: 1031.4474. ^{1}H NMR (500 MHz, DMSO-d$_6$) δ 8.02 (dd, *J* = 5.8, 3.3 Hz, 2H), 7.91 (dd, J = 5.8, 3.3 Hz, 2H), 7.62 (t, *J* = 7.8 Hz, 2H), 7.52 (s, 1H), 7.34 (d, *J* = 7.8 Hz, 4H), 7.26 (s, 1H), 6.82 (s, 2H), 3.81 – 3.70 (m, 4H), 3.14 (q, *J* = 6.3 Hz, 2H), 2.78 (t, *J* = 6.7 Hz, 2H), 2.58 – 2.51 (m, 4H), 2.40 (s, 3H), 1.56 (s, 6H), 1.04 (dd, *J* = 6.9, 3.7 Hz, 24H), ^{13}C NMR (126 MHz, DMSO) δ 180.16, 174.39, 155.20, 144.61, 138.33, 134.47, 133.62, 133.47, 132.82, 132.46, 131.87, 130.65, 129.06, 126.13, 123.86, 123.33, 62.50, 49.28, 39.95, 39.85, 39.78, 39.69, 39.61, 39.52, 39.45, 39.35, 39.28, 39.19, 39.02, 38.85, 28.88, 27.76, 23.97, 23.40, 20.68, 16.56.

Oxali^{IV}(Gem)(Au(I)) – Gen 1

This Pt(IV)-Au(I) complex was prepared using a reported procedure.[2]

^{1}H NMR (500 MHz, DMSO-*d$_6$*) δ 8.03 (dd, *J* = 5.7, 3.3 Hz, 2H), 7.91 (dd, *J* = 5.8, 3.3 Hz, 2H), 7.63 (t, *J* = 7.8 Hz, 2H), 7.51 (s, 1H), 7.35 (d, *J* = 7.8 Hz, 4H), 7.06 (s, 2H), 6.82 (s, 2H), 3.83 – 3.71 (m, 7H), 2.97 – 2.92 (m, 5H), 2.91 – 2.83 (m, 2H), 2.54 (q, *J* = 6.0 Hz, 6H), 2.41 (s, 3H), 2.16 – 1.99 (m, 2H), 1.56 (s, 6H), 1.49 (t, *J* = 15.4 Hz, 4H), 1.32 (s, 1H), 1.05 (dd, *J* = 6.8, 4.3 Hz, 25H). ^{195}Pt NMR (108 MHz, DMSO-*d$_6$*) δ 1417.35. HPLC – 6.03 min (0-100% ACN in 5.84 min + 2 min constant 100% ACN) ESI-HRMS (*m/z*) calculated for $[C_{61}H_{76}AuN_8O_{11}Pt]^+$: 1489.37, found 1489.49;

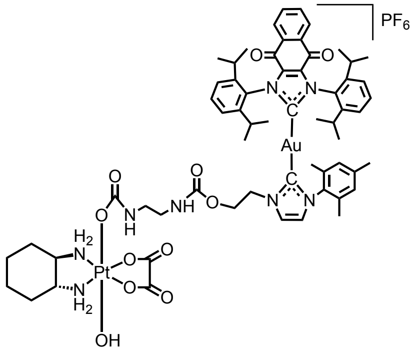

Pt^{IV}-Au^I Gen 1

cct-[Pt((1R,2R)-DACH)(ox)(Gem-Bis BOC protected)(OH)] (**5**)

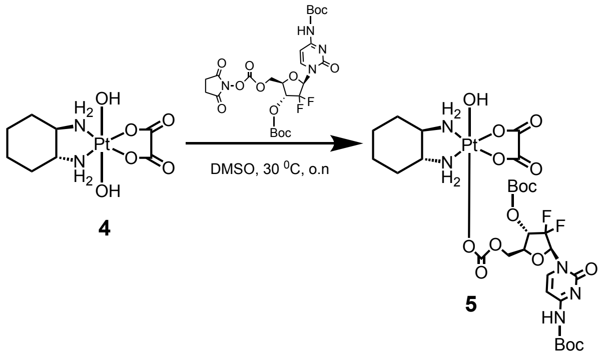

98.5 mg of **4** were dissolved in 10mL of DMSO and 165.7 mg (1.2 equiv.) of gem protected-DSC[3] were added. The reaction mixture was stirred overnight at 30ºC. Afterwards, the presumed product was collected through ether precipitation and washed several times with ether. Yield – 75%, 158 mg. ^{195}Pt NMR (108 MHz, DMSO) δ 1416.64. HPLC – 4.81 min (0-100% ACN in 5.84 min + 2 min constant 100% ACN)

cct-[Pt((1R,2R)-DACH)(ox)(gemcitabine)(OH)] (Oxali(Gem)(OH) (**6**)

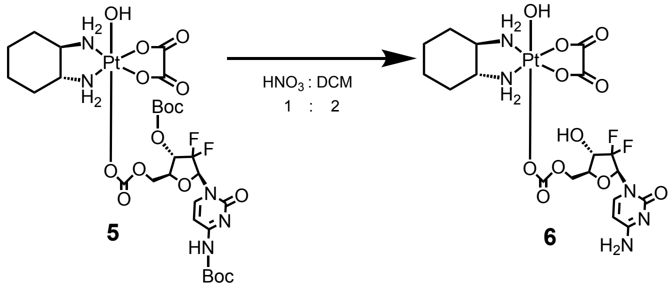

220 mg of **5** were stirred in 6 mL of a 2:1 DCM : HNO_3 mixture for 10 min until a clear solution was formed. Afterwards, the volatiles were evaporated off under reduced pressure and the product was precipitated using methanol and diethyl ether to give 163.8 mg of a grey color product. Yield 95%. HPLC – 2.91 min (0-100% ACN in 5.84 min + 2 min constant 100% ACN)

cct-[Pt((1R,2R)-DACH)(ox)(gemcitabine)(succinimidyl carbonate)] (**7**)

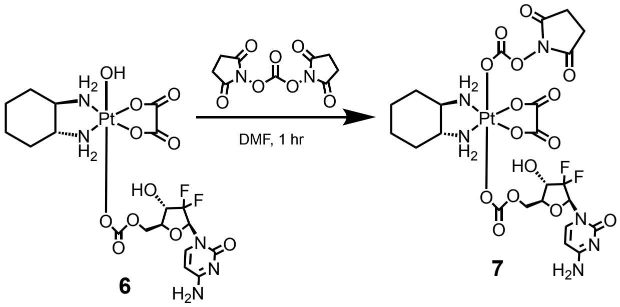

115 mg of **6** were dissolved in 3mL of DMF and 204 mg (5 equiv.) of N,N′-Disuccinimidyl carbonate was added for 1 h. Reaction was monitored via HPLC. Once reaction was done, solvent was removed under reduced pressure and product was extracted using ACN and diethylether. Yield – 80%, 110 mg. ^{195}Pt NMR (108 MHz, DMSO) δ 1684.71. HPLC – 3.00 min (0-100% ACN in 5.84 min + 2 min constant 100% ACN)

Oxali^{IV}(Gem)(Au(I)) – Gen 2

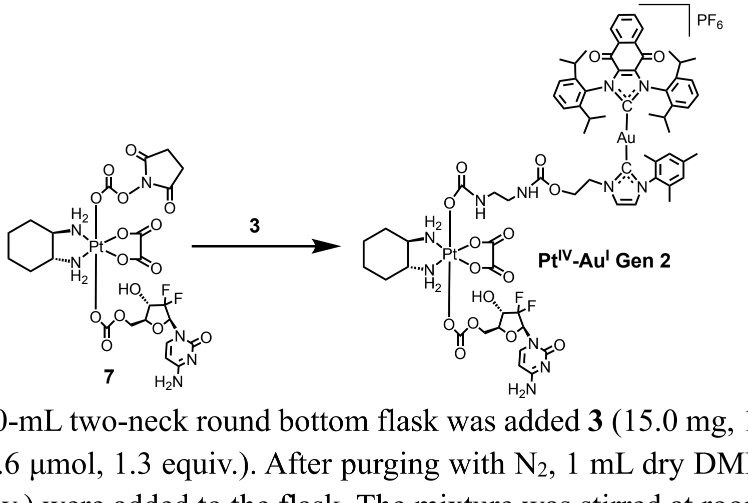

To an oven-dried 10-mL two-neck round bottom flask was added **3** (15.0 mg, 12.7 µmol, 1 equiv.) and **7** (14.3 mg, 16.6 µmol, 1.3 equiv.). After purging with N_2, 1 mL dry DMF and TEA (4.6 µL, 33.1 µmol, 2.6 equiv.) were added to the flask. The mixture was stirred at room temperature for 4 h. Boc2O (5.9 µL, 25.5 µmol, 2 equiv.) and additional TEA (3.6 µL, 25.5 µmol, 2 equiv.) were then added to the flask and the mixture was further stirred at room temperature for 2 h. The volatiles

were evaporated off under reduced pressure and the remaining mixture was re-dissolved in 400 µL MeOH. The mixture was purified using a semi-preparative RP-HPLC. Solvent was evaporated under reduced pressure and the resulting mixture subject to PF_6^- anion exchange chromatography. The aqueous suspension obtained in this way was lyophilized for 48 h to afford a light-yellow solid (8.8 mg, 33%). ^{1}H NMR (600 MHz, DMSO-d_6) δ 8.00 (dt, J = 7.4, 3.7 Hz, 2H), 7.89 (dd, J = 5.8, 3.3 Hz, 2H), 7.61 (t, J = 7.8 Hz, 2H), 7.50 (s, 1H), 7.48 (s, 1H), 7.40 (s, 1H), 7.35 (s, 1H), 7.32 (d, J = 8.0 Hz, 5H), 7.05 (s, 1H), 6.85 (d, J = 6.0 Hz, 1H), 6.79 (s, 2H), 6.14 (s, 1H), 5.77 (d, J = 7.5 Hz, 1H), 4.30 – 3.85 (m, 4H), 3.71 (s, 3H), 2.91 (s, 4H), 2.57 (s, 3H), 2.55 – 2.48 (m, 3H), 2.38 (s, 4H), 1.54 (s, 6H), 1.48 (s, 6H), 1.12 (d, J = 9.1 Hz, 0H), 1.02 (dd, J = 6.8, 4.8 Hz, 25H). ^{13}C NMR (151 MHz, DMSO-d_6) δ 192.93, 180.75, 174.96, 166.09, 158.05, 155.82, 155.11, 154.98, 145.21, 138.91, 135.05, 134.22, 134.07, 133.41, 133.05, 132.46, 131.25, 129.64, 126.72, 124.45, 123.48, 118.75, 95.30, 94.99, 80.85, 77.88, 66.48, 62.59, 62.25, 60.09, 58.97, 49.32, 40.40, 40.26, 40.12, 39.98, 39.84, 39.70, 39.56, 33.28, 31.73, 31.38, 29.22, 29.14, 28.36, 24.54, 23.97, 23.30, 22.53, 21.25, 17.13.^{195}Pt NMR (108 MHz, DMSO) δ 1601.46. HPLC – 5.70 min (0-100% ACN in 5.84 min + 2 min constant 100% ACN). ESI-HRMS (acetonitrile) (m/z): calculated for $C_{71}H_{85}AuF_2N_{11}O_{16}Pt$: 1778.57, observed: 1778.54.

1.3. Reduction and Stability

Second generation Pt(IV)-Au(I) complex was dissolved in MeOH. Concentrated solutions of ascorbic acid, GSH or mixture of both GSH and ascorbic acid were prepared in water, and the pH was adjusted to 7.0 with NaOH. A 10-fold molar excess of the corresponding solution was then added and reduction was monitored by analytical HPLC. The half-lives ($t_{1/2}$) for reduction was determined via linear fitting of $\ln(A_t/A_0)$ vs time (t) in accord with the pseudo first order rate equation $A_t = A_0e^{-kt}$, where A_0 and A_t are the integrated areas of the HPLC peaks of the respective complexes at t = 0 and at time t, respectively, and k (slope) is the rate constant. The value of $t_{1/2}$ was calculated using the equation $t_{1/2} = -\ln(2)/k$.

Stability studies were conducted by dissolving the complex in 1% DMSO and RPMI medium at pH 7.4, and maintaining them at 37°C. HPLC chromatograms were recorded until the peak areas were less than half the starting peak area. The $t_{1/2}$ value was calculated by linear fitting of $\ln(A_t/A_0)$ vs time (t) where A_t and A_0 are the integrated peak areas determined by HPLC at t = 0 and at time t.

1.4. Antiproliferative assay in tumorigenic cells

Cell proliferation studies: A549 (ATCC®) cells and CT26 (ATCC®) cells were harvested and seeded into 96-well culture plates (Costar 07-200-90) in 100 µL of culture medium containing 10% fetal bovine serum (FBS) and 2% penicillin/streptomycin (P/S). The cells were allowed to incubate overnight at 37 ºC in the presence of 5% CO_2. A549 cells were seeded at a density of 1500 cells/well and CT26 at a density of 750 cells/well. The next day, appropriate serial dilutions of drug stocks in culture media were made. To each well of a 96 well plate was added 100 µL of the appropriate solution. After a total of three days, a 50 µL aliquot of 3 mg/mL tetrazolium dye, 3-(4,5-dimethylthiazol-2-yl)-2,5diphenyltetrazolium bromide (Alfa Aesar L11939), dissolved in

culture medium without FBS was added to each well, followed by a 4 h incubation period at 37 ºC. After removal of the medium, the resulting formazan crystals were dissolved in 50 µL DMSO and the absorbance was measured at 560–650 nm using a microplate reader (Molecular Devices, Sunnyvale, CA). Absorbance values were corrected for background and then normalized to wells containing untreated cells. The resulting dose response curves were subjected to linear regression analysis using Origin (OriginLab, Inc.) to determine the IC_{50} values. Data are shown as mean inhibition of proliferation or growth as a percentage of control cells and are from 3 replicate experiments. The maximum concentration of DMSO used was 0.1%. This concentration of DMSO was determined to be non-toxic in separate control experiments.

1.5. Total Cellular Metal Accumulation

1 x 10^6 CT26 cells were seeded in a 6-well plate (2 mL RPMI-1640) containing 10% FBS and 2% P/S. After incubating overnight, the cell media was replaced, with new media containing test compounds (1 µM) for 24 h. After 24 hours, cell monolayers were washed twice with cold PBS, harvested and counted in PBS for a total volume of 400 µL. Samples were divided, and each was mineralized with 1 mL aqua regia and evaporated to dryness on a 110 ºC hot plate overnight. Later, 1 mL of 2% HCl + 1% thiourea was added to each vial. Samples were quantified via ICP-MS. Metal accumulation levels were determined using the relative isotopic prevalence of ^{195}Pt and ^{197}Au. Experiments were performed in triplicates (n = 3), and data are reported as mean ± SD. Statistical analysis was performed using a two-way ANOVA followed by Tukey's post hoc test.

1.6. Metal Accumulation in Organelles

10 x 10^6 CT26 cells were seeded in T-150 flasks (50 mL RPMI-1640) containing 10% FBS and 2% P/S. After incubating overnight, the medium was replaced, and the cells were treated with either a 1:1:1 mixture of Au(I)-amine + oxaliplatin + gemcitabine or Pt(IV)-Au(I) Gen 2 at 1 µM concentration for 24 h. The resulting cell monolayers were washed twice with cold PBS, harvested with trypsin, counted and resuspended in PBS for a total volume of 1000 µL. Isolation of nuclei, cytoplasm, and mitochondria was carried out using a reported procedure.[4] Samples were quantified via ICP-MS. Metal accumulations were determined using the relative isotopic prevalence of ^{195}Pt and ^{197}Au. Experiments were performed in triplicates (n = 3), and statistical analysis was carried out using a two-way ANOVA followed by Tukey's post hoc test.

1.7. Extracellular ATP release

Extracellular ATP was quantified following the RealTime-Glo Extracellular ATP protocol (Promega catalogue number GA5010) after 24 h exposure of CT-26 cells to either oxaliplatin (150 µM), gemcitabine (0.5 µM) or Pt(IV)-Au(I) Gen 2 (1.25 µM). Experiments were performed in triplicates (n = 3), and statistical analysis was carried out using a one-way ANOVA followed by Tukey's post hoc test.

1.8. HMGB1 release

HMGB1 release was quantified using Mouse HMGB-1 ELISA kit (Invitrogen catalogue number EEL102) after 24 h exposure of CT-26 cells to either oxaliplatin (150 µM), gemcitabine (0.5 µM)

or Pt(IV)-Au(I) Gen 2 (1.25 μM). Experiments were performed in triplicates (n = 3), and statistical analysis was carried out using a one-way ANOVA followed by Tukey's post hoc test.

1.9. CRT Translocation Confocal Microscopy

CT26 cells were seeded at a density of 3 x 10^5 cells/dish overnight in 35 mm dishes with a 10 mm glass diameter containing a poly-D lysine coat (Mat Tek P35GC-1.5- 10-C). Cells were then incubated overnight at 37 ˚C in the presence of 5% CO2. After overnight incubation, media was removed and each dish was given new media with the respective treatments (oxaliplatin - 300 μM, gemcitabine - 1 μM, Pt(IV)-Au(I) Gen 2 - 2.5 μM). Untreated group received media only. Cells were incubated for another 4 hours at 37 ºC. Post incubation, media was removed, and cells were washed (3x) with HBSS. Cells were then fixed with 4% paraformaldehyde for 20 min at RT. After fixation cells were washed (3x) with HBSS. For staining, cells were incubated with CRT polyclonal antibody (Invitrogen PA3-900) in cell staining buffer (Biolegend 420201) at a 1:200 dilution for 1 h on ice. Cells were washed (3x) with HBSS and placed in the dark. Next, cells were stained with goat anti-rabbit IgG Alexa Fluor 488 antibody (Invitrogen A-110 08) in cell staining buffer at a 1:500 dilution for 30 min on ice. Cells were washed (3x) with HBSS stained with wheat germ agglutinin Alexa Fluor 633 5 μg/mL (Invitrogen W21404) in PBS for 5 min at RT and directly imaged fluorescently on a Leica SP5 X white light laser confocal microscope. Images were taken with a 63X, NA 1.4 objective.

2.0. RNA Sequencing (RNA-Seq)

CT26 cells were seeded at a density of 1 x 10^6 cells/10 mL in a T75 tissue culture flask. All cells came from the same starting flask. Once seeded, flasks were incubated for 24 hours. After 24 hours old media was removed from each flask and 10 mL of fresh media containing each compound at the IC_{50} concentration was added. For the control flask only 10 mL of fresh media was added (untreated). After 4 hour incubation at 37 ºC in the presence of 5% CO_2, the media was removed from each flask and the cells were washed with warm PBS and treated with trypsin. The trypsin was quenched with media and cells were transferred to 15 mL centrifuge tubes where they were pelleted. The media was discarded, and the cell pellet was washed 3x with PBS. Cells were then re-suspended in PBS and treated with QIAzol Lysis Reagent and homogenized by vigorous vortexing and shaking. The QIAGEN RNeasy Plus Universal Mini Total RNA protocol was then picked up at Step 4. RNA was eluted in two volumes of 30 μL each for a final volume of 60 μL RNA. RNA concentration was measured using a Thermo Scientific NanoDrop 2000c Spectrophotometer. The Agilent TapeStation system was used to ensure the integrity of the RNA through visualization of ribosomal subunits. Samples were submitted for microarray analysis, from 3 independent experiments to the Interdisciplinary Center for Biotechnology Research at the University of Florida. Common genes between different comparisons within three biological replicates were analyzed based on the cutoffs (abs(log2(FC)) ≥ 1, and the FDR-corrected P-value ≤ 0.05. Analysis settings for Ingenuity Canonical Pathways: Cutoff before duplicate resolution = True, Resolve duplicate on Expression log fold change**,** Color using Expression log fold change**,**

Cutoff for Expression log fold change down regulated -1 and up regulated 1.0, Cutoff for Expression value 0.05, Reference set Ingenuity Knowledge Base (Genes Only).

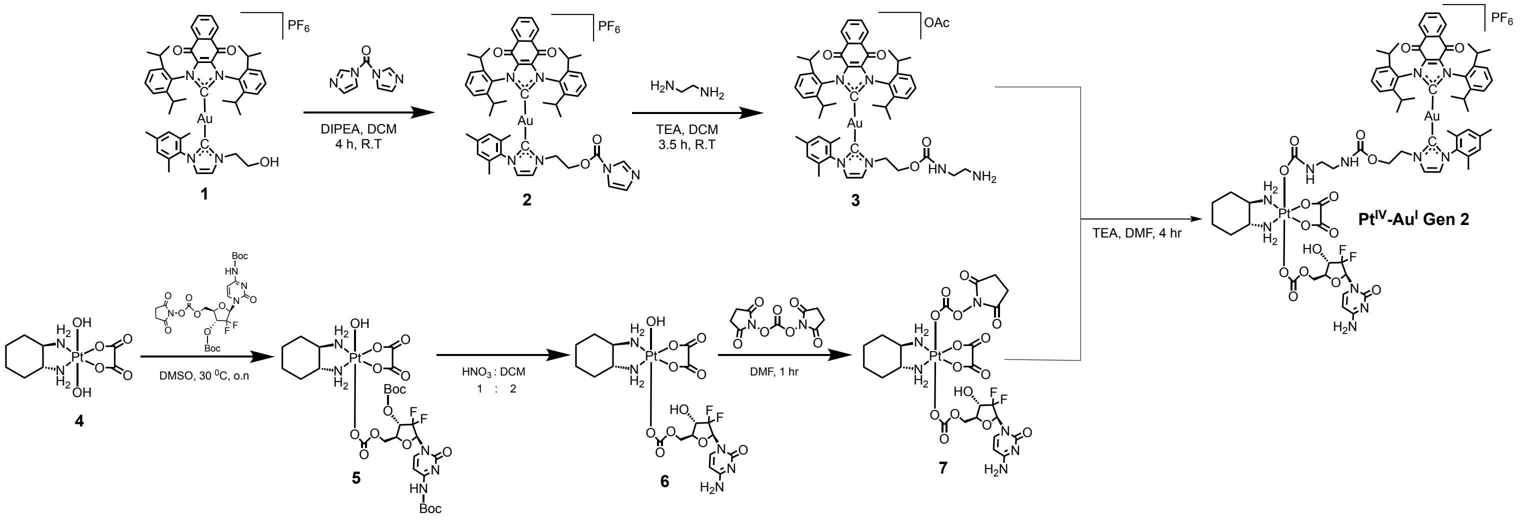

Scheme 1 Synthetic route to Oxali(Gem)(Au(I)) – Gen 2

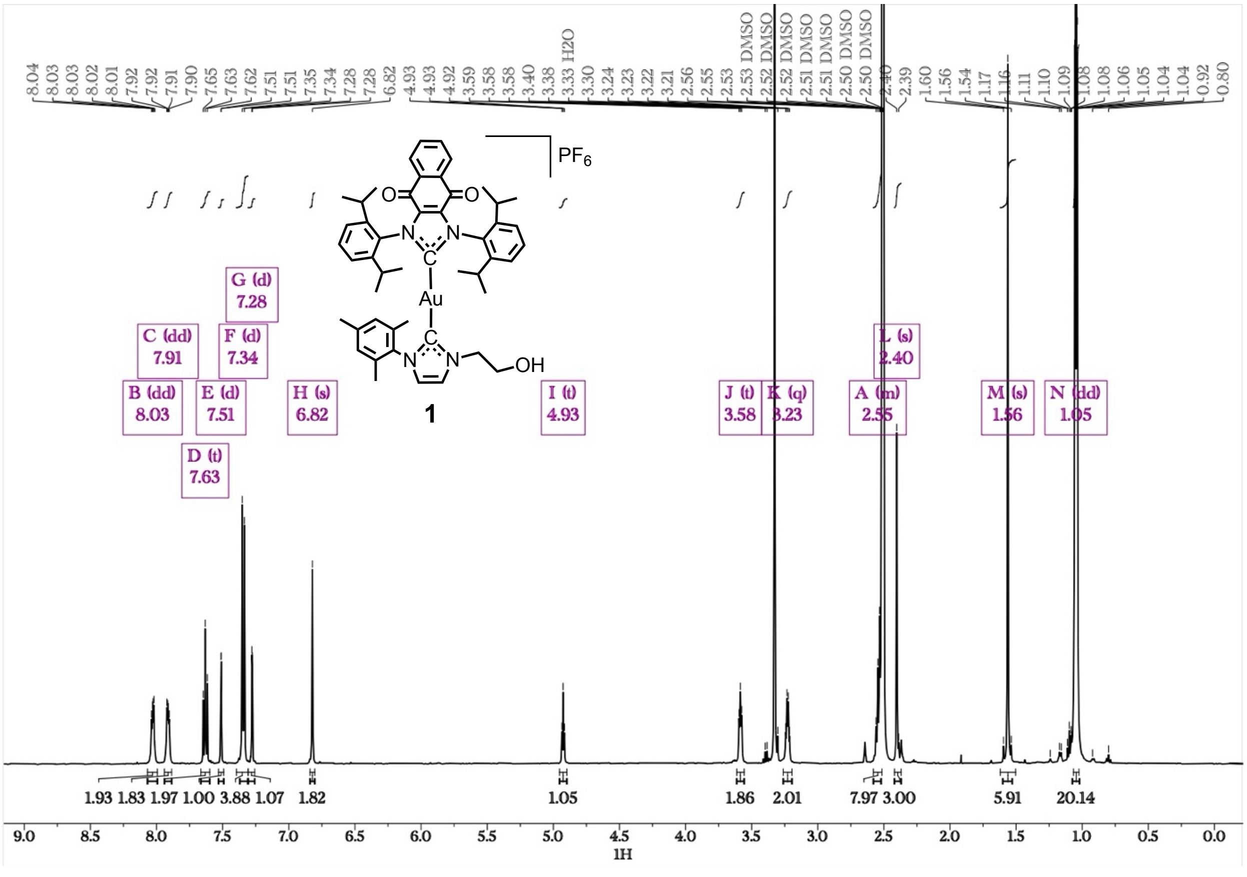

Figure S1 ^{1}H NMR spectrum of (1,3-bis(2,6-diisopropylphenyl)-4,9-dioxo-1,3,4,9-tetrahydro-2*H*-2λ3-naphtho[2,3-*d*]imidazol-2-yl)(1-(2-hydroxyethyl)-3-mesityl-1,3-dihydro-2*H*-2λ3-imidazol-2-yl) gold(I) in DMSO-d_6

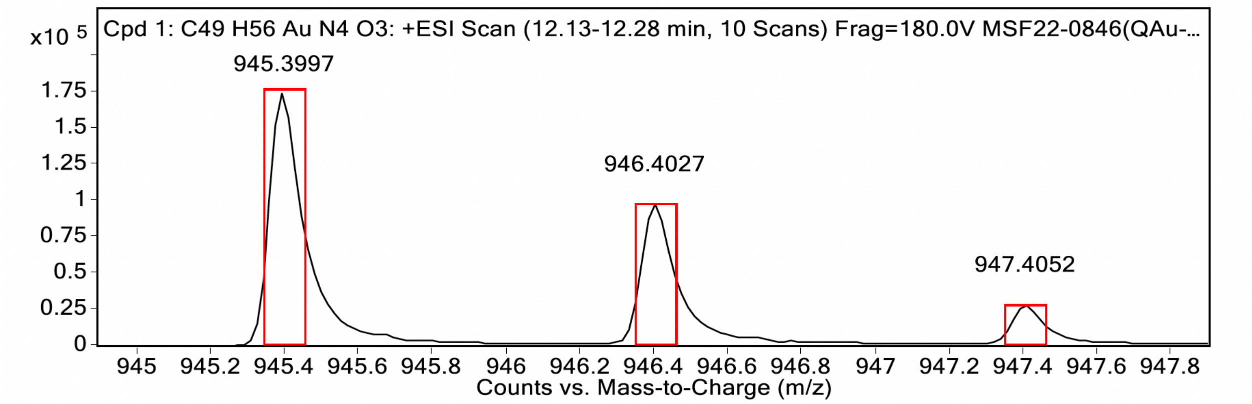

Figure S2 HRMS (1,3-bis(2,6-diisopropylphenyl)-4,9-dioxo-1,3,4,9-tetrahydro-2*H*-2λ3-naphtho[2,3-*d*]imidazol-2-yl)(1-(2-hydroxyethyl)-3-mesityl-1,3-dihydro-2*H*-2λ3-imidazol-2-yl) gold(I)

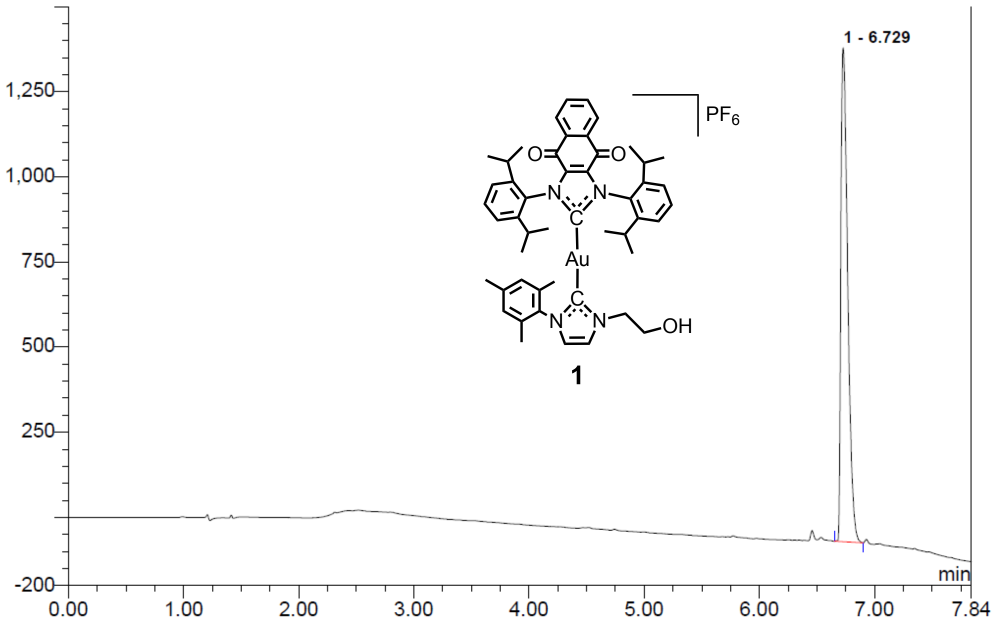

Figure S3 HPLC chromatogram of (1,3-bis(2,6-diisopropylphenyl)-4,9-dioxo-1,3,4,9-tetrahydro-2*H*-2λ3-naphtho[2,3-*d*]imidazol-2-yl)(1-(2-hydroxyethyl)-3-mesityl-1,3-dihydro-2*H*-2λ3-imidazol-2-yl) gold(I) - 0-100% acetonitrile in 5.84 min + 2 min constant 100% acetonitrile.

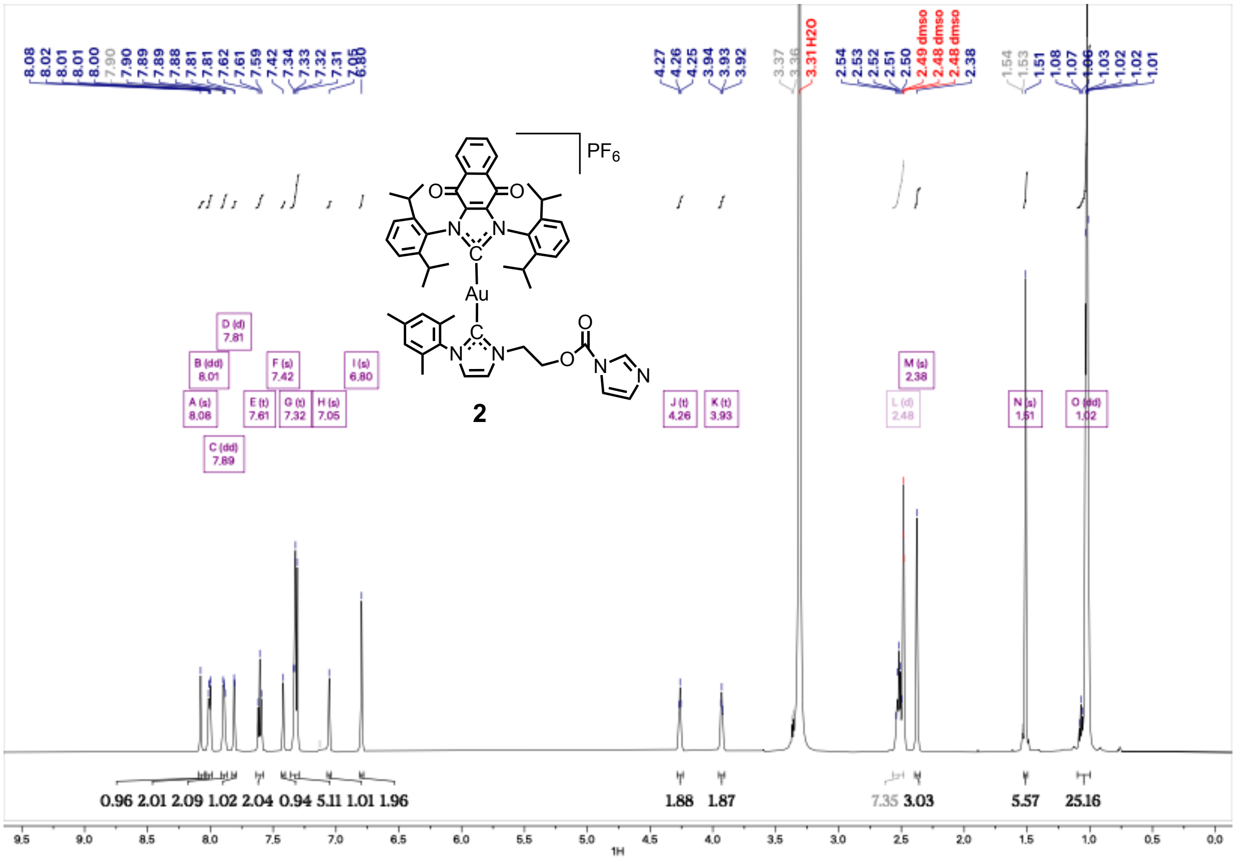

Figure S4 ^{1}H NMR spectrum of (1-(2-((1*H*-imidazole-1-carbonyl)oxy)ethyl)-3-mesityl-1,3-dihydro-2*H*-2λ3-imidazol-2-yl)(1,3-bis(2,6-diisopropylphenyl)-4,9-dioxo-1,3,4,9-tetrahydro-2*H*-2λ3-naphtho[2,3-*d*]imidazol-2-yl) gold(I) in DMSO-d_6

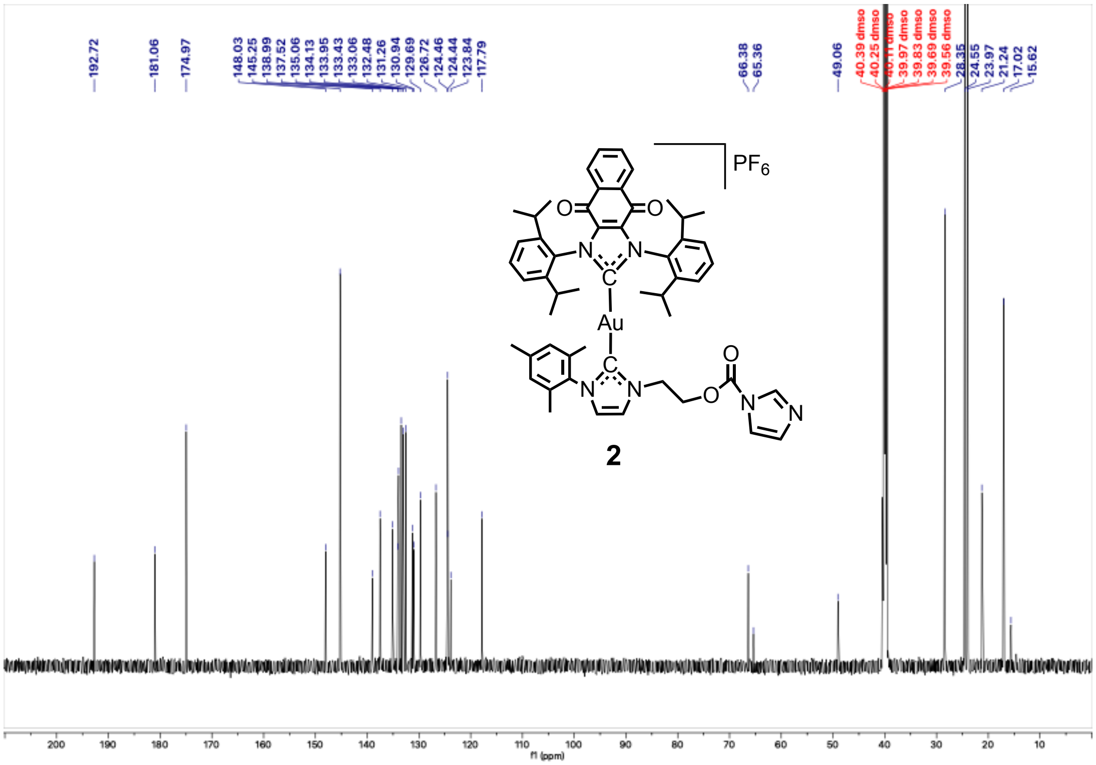

Figure S5 ^{13}C NMR spectrum of (1-(2-((1*H*-imidazole-1-carbonyl)oxy)ethyl)-3-mesityl-1,3-dihydro-2*H*-2λ3-imidazol-2-yl)(1,3-bis(2,6-diisopropylphenyl)-4,9-dioxo-1,3,4,9-tetrahydro-2*H*-2λ3-naphtho[2,3-*d*]imidazol-2-yl) gold(I) in DMSO-d_6

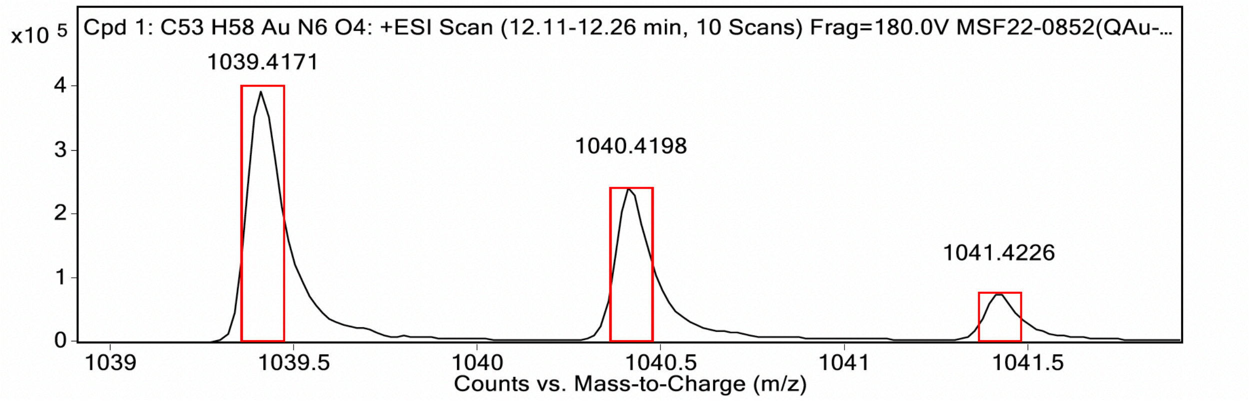

Figure S6 HRMS of (1-(2-((1*H*-imidazole-1-carbonyl)oxy)ethyl)-3-mesityl-1,3-dihydro-2*H*-2λ3-imidazol-2-yl)(1,3-bis(2,6-diisopropylphenyl)-4,9-dioxo-1,3,4,9-tetrahydro-2*H*-2λ3-naphtho[2,3-*d*]imidazol-2-yl) gold(I)

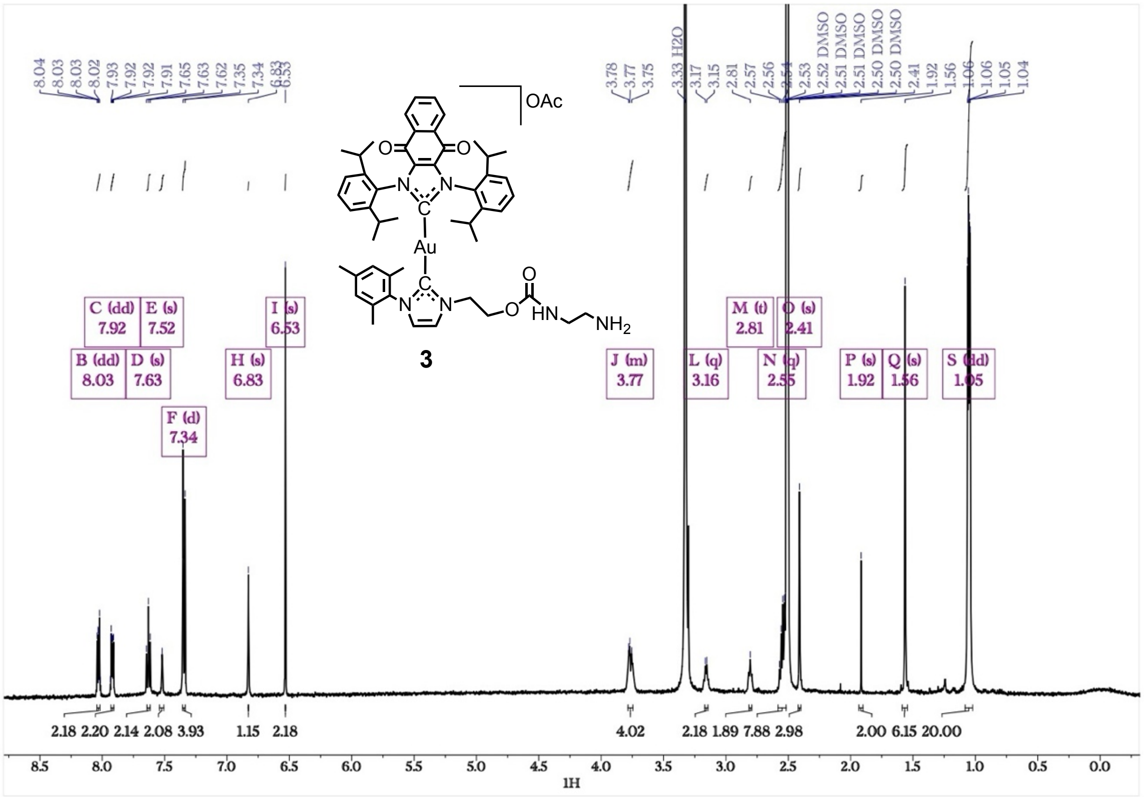

Figure S7 ^{1}H NMR spectrum of (1-(2-(((2-Aminoethyl)carbamoyl)oxy)ethyl)-3-mesityl-1,3-dihydro-2H-2λ3-imidazol-2-yl)(1,3-bis(2,6-diisopropylphenyl)-4,9-dioxo-1,3,4,9-tetrahydro-2H-2λ3-naphtho[2,3-d]imidazol-2-yl)gold (I) in DMSO-d_6

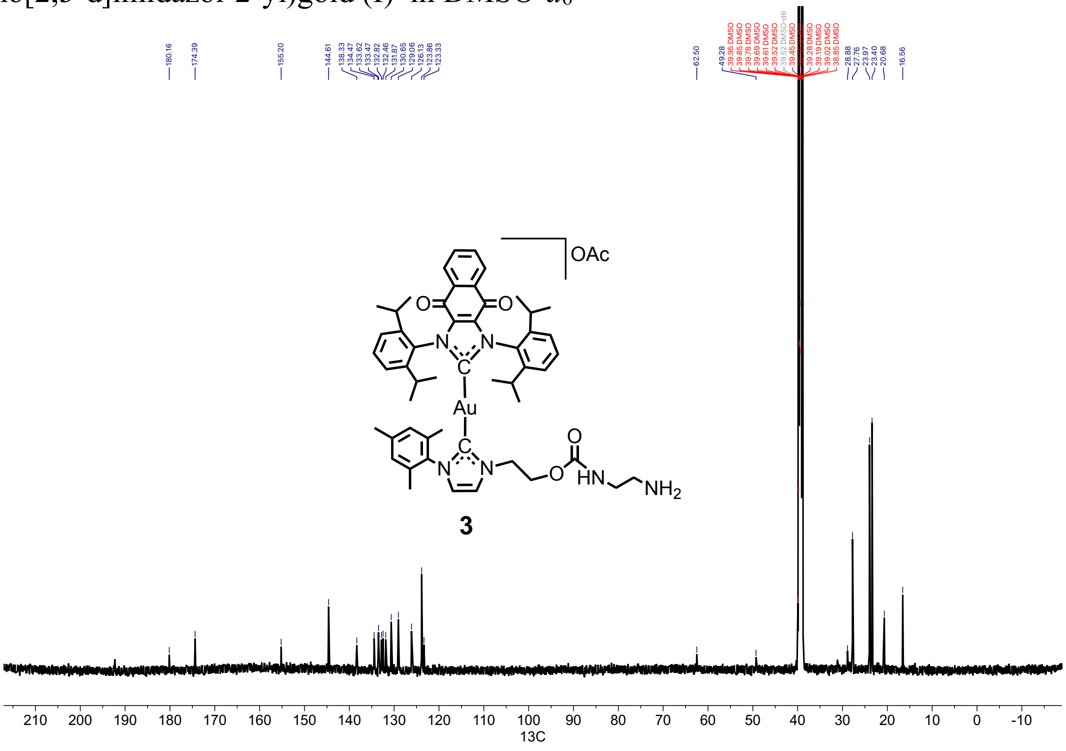

Figure S8 ^{13}C NMR spectrum of (1-(2-(((2-Aminoethyl)carbamoyl)oxy)ethyl)-3-mesityl-1,3-dihydro-2H-2λ3-imidazol-2-yl)(1,3-bis(2,6-diisopropylphenyl)-4,9-dioxo-1,3,4,9-tetrahydro-2H-2λ3-naphtho[2,3-d]imidazol-2-yl)gold (I) in DMSO-d_6

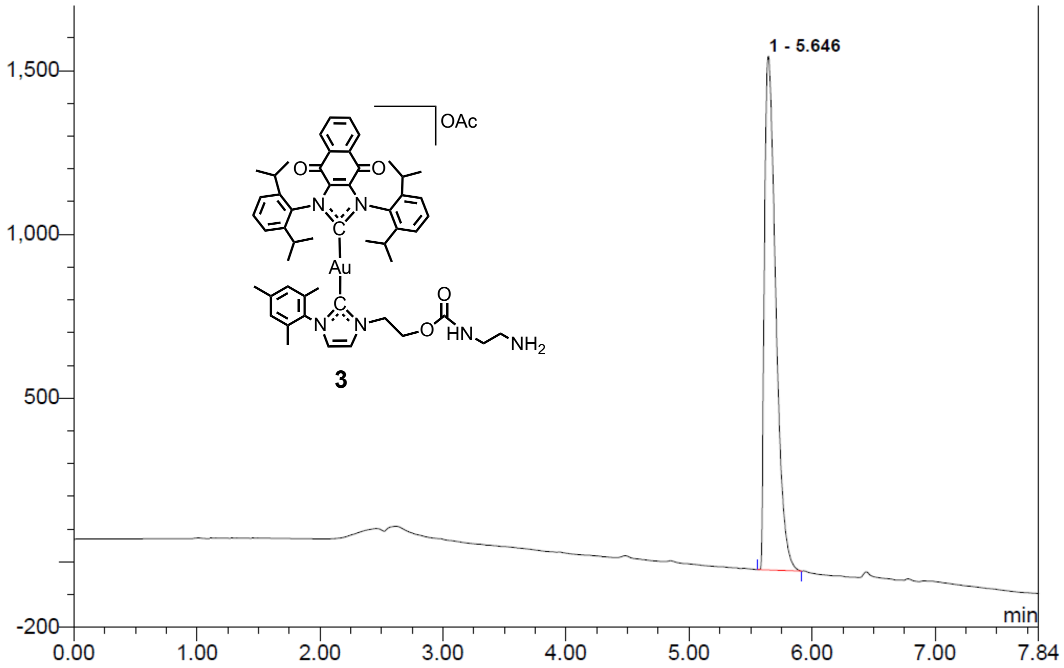

Figure S9 HPLC chromatogram of (1-(2-(((2-Aminoethyl)carbamoyl)oxy)ethyl)-3-mesityl-1,3-dihydro-2H-2λ3-imidazol-2-yl)(1,3-bis(2,6-diisopropylphenyl)-4,9-dioxo-1,3,4,9-tetrahydro-2H-2λ3-naphtho[2,3-d]imidazol-2-yl)gold (I) - 0-100% acetonitrile in 5.84 min + 2 min constant 100% acetonitrile.

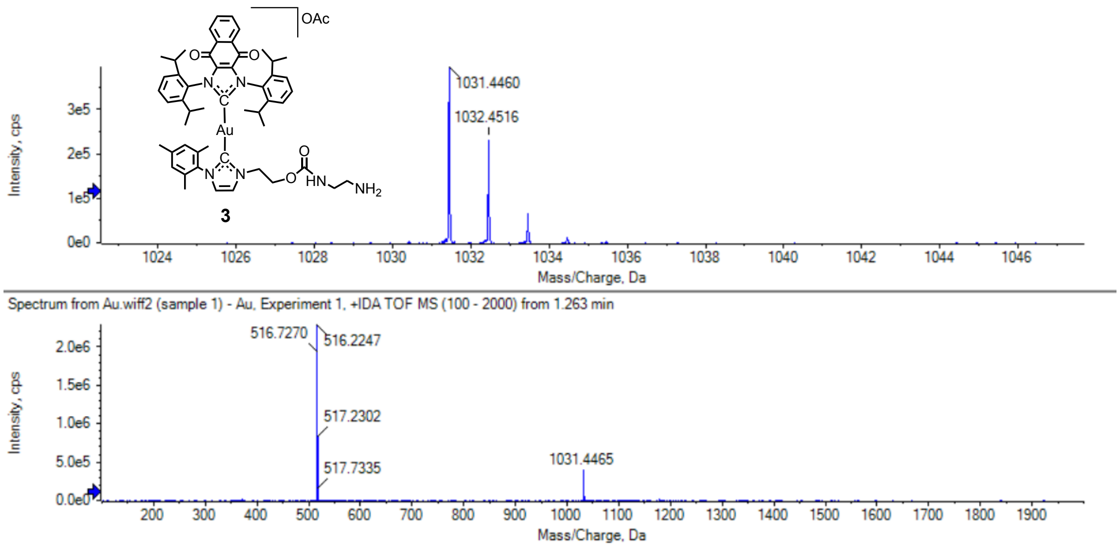

Figure S10 ESI-HRMS (+ve) of (1-(2-(((2-Aminoethyl)carbamoyl)oxy)ethyl)-3-mesityl-1,3-dihydro-2H-2λ3-imidazol-2-yl)(1,3-bis(2,6-diisopropylphenyl)-4,9-dioxo-1,3,4,9-tetrahydro-2H-2λ3-naphtho[2,3-d]imidazol-2-yl)gold (I)

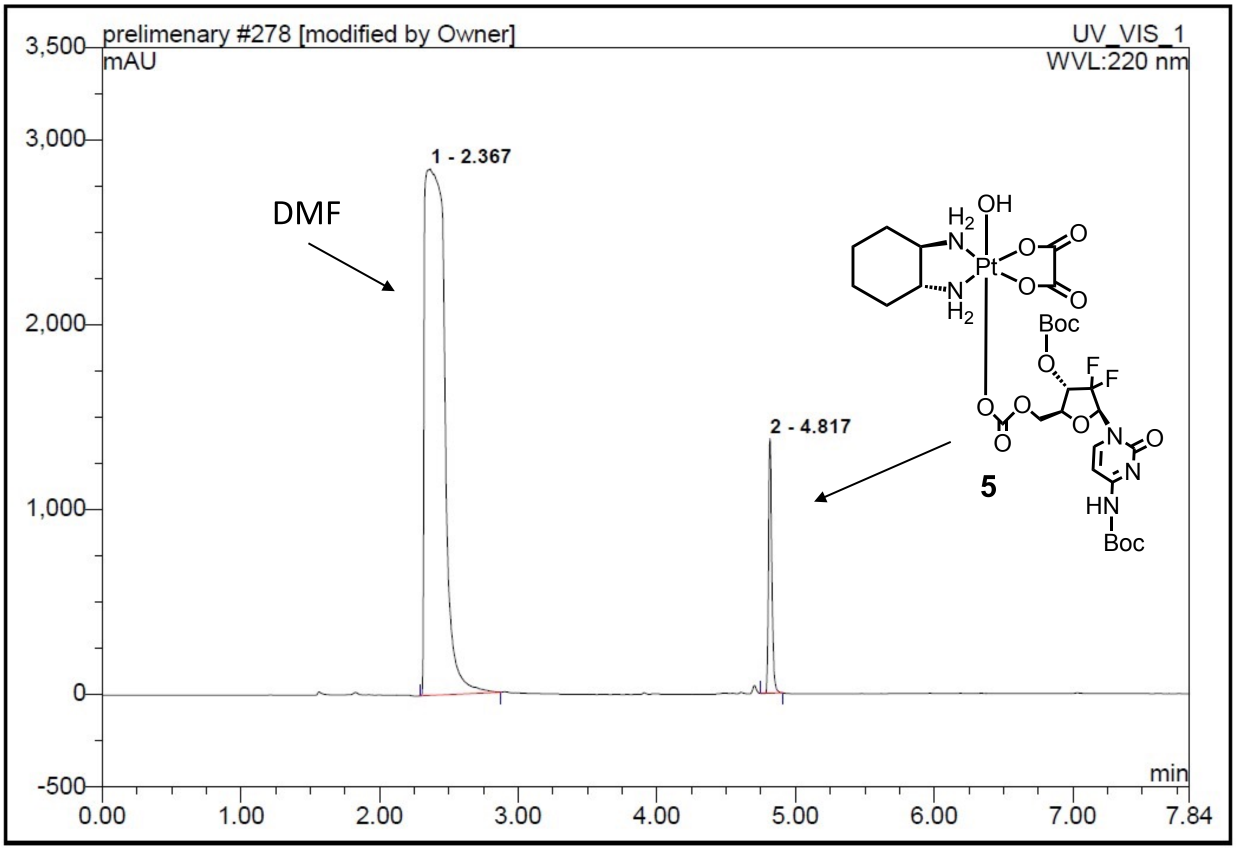

Figure S11 HPLC chromatogram of Oxali(Gem-Bis BOC protected)(OH)- 0-100% acetonitrile in 5.84 min + 2 min constant 100% acetonitrile.

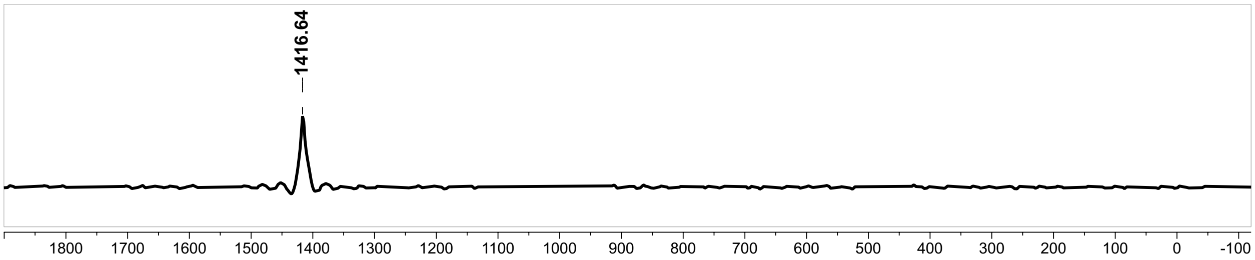

Figure S12 ^{195}Pt NMR spectrum of Oxali(Gem-Bis BOC protected)(OH)

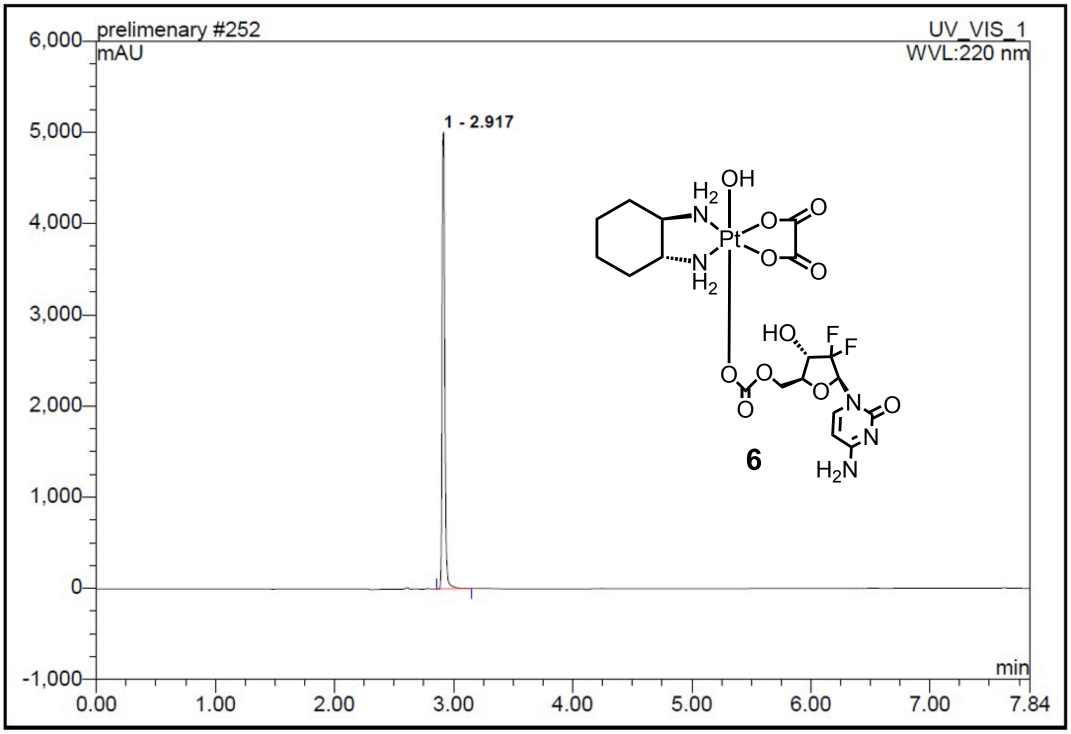

Figure S13 HPLC chromatogram of Oxali(Gem)(OH)- 0-100% acetonitrile in 5.84 min + 2 min constant 100% acetonitrile.

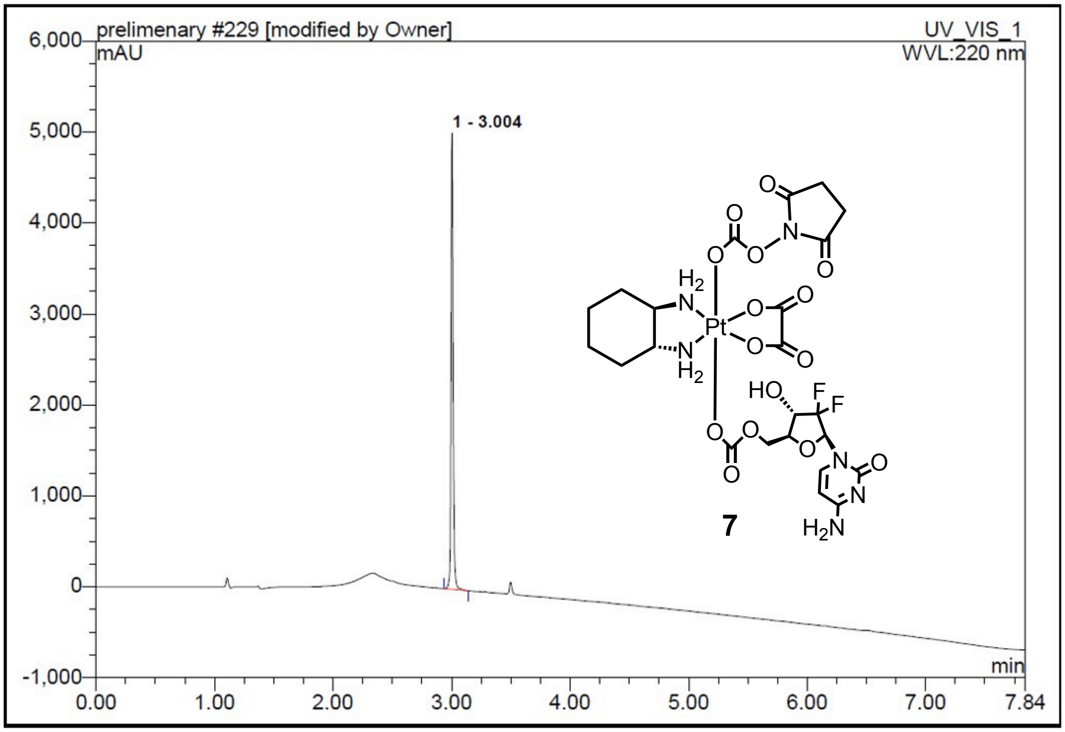

Figure S14 HPLC chromatogram of Oxali(Gem)(MSC)- 0-100% acetonitrile in 5.84 min + 2 min constant 100% acetonitrile.

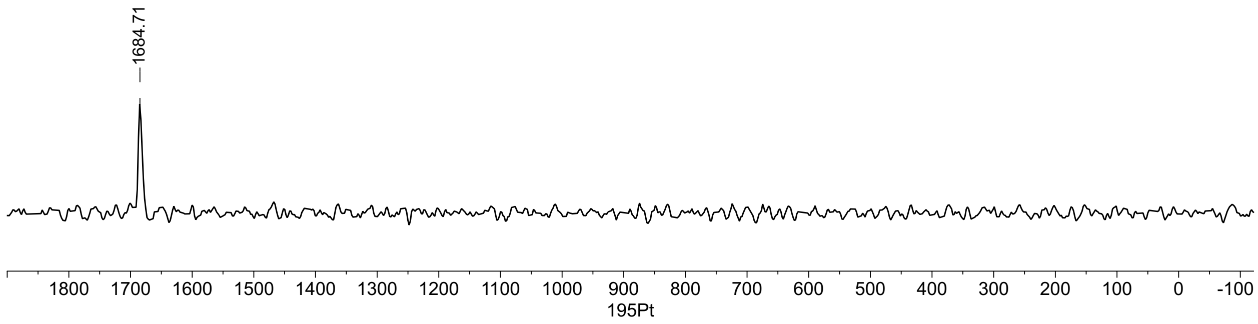

Figure S15 ^{195}Pt NMR spectrum of Oxali(Gem)(MSC)

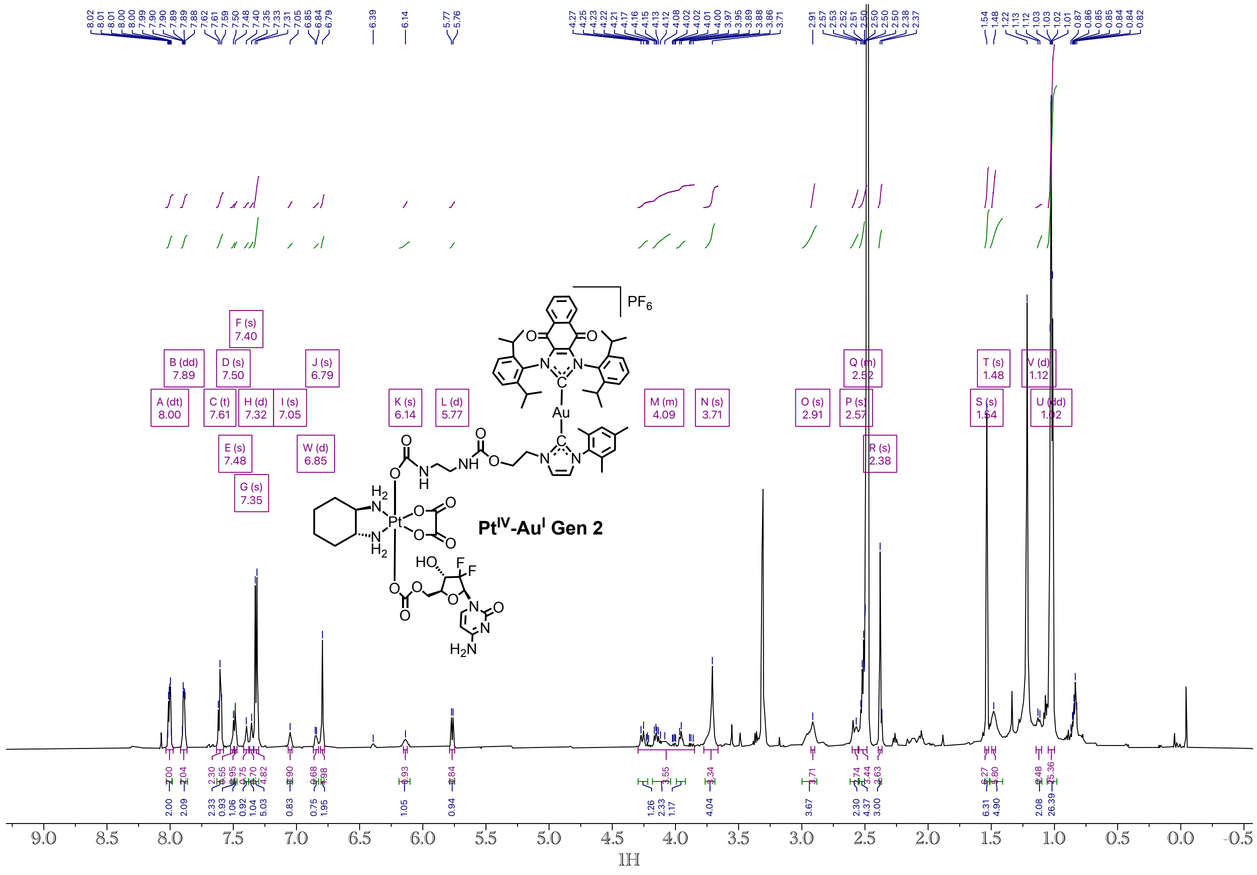

Figure S16 ^{1}H NMR spectrum of Oxali(Gem)(Au(I)) – Gen 2 in DMSO-d_6

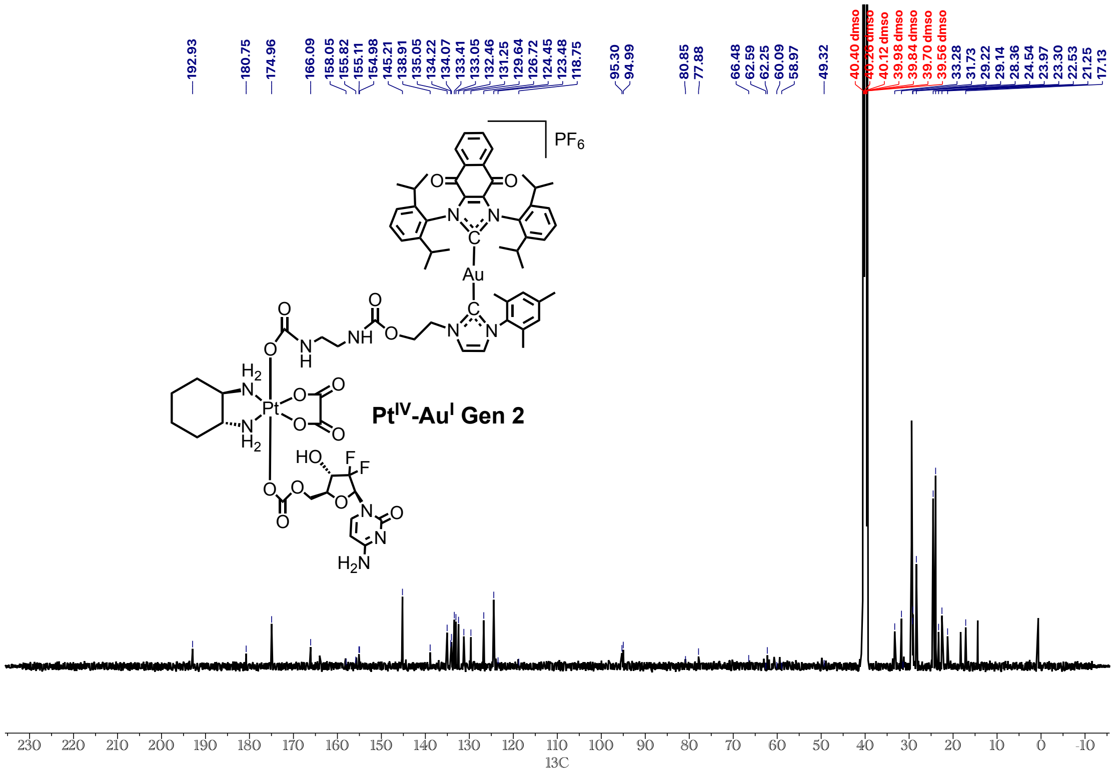

Figure S17 ^{13}C NMR spectrum of Oxali(Gem)(Au(I)) – Gen 2 in DMSO-d_6

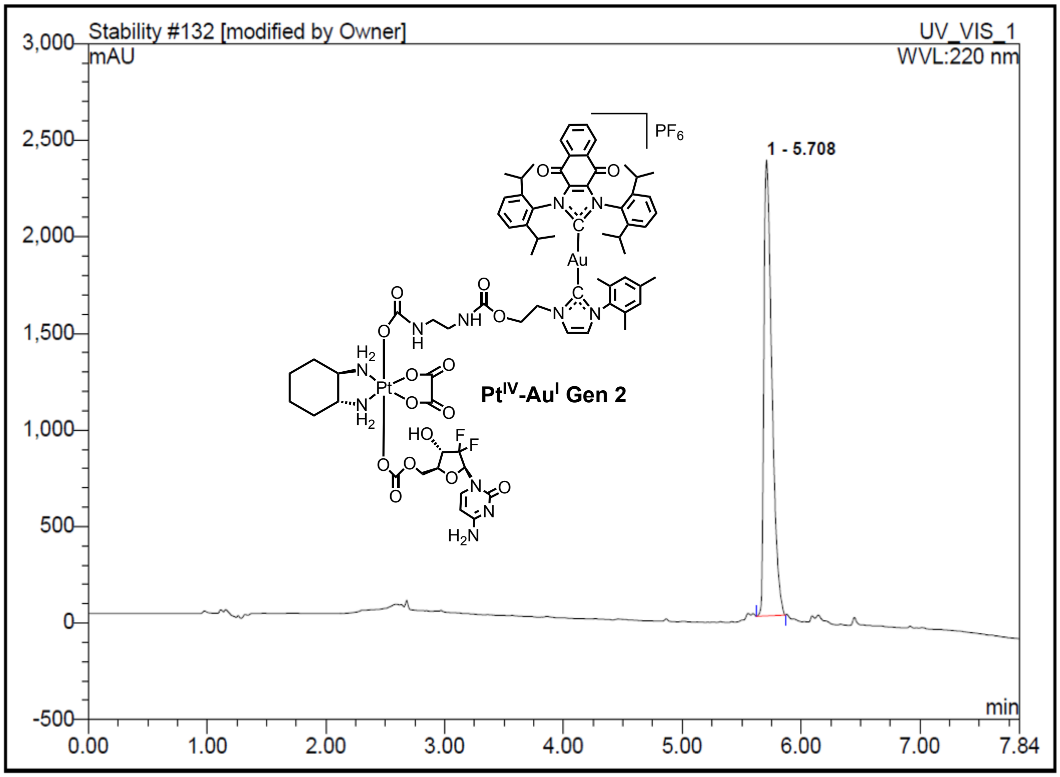

Figure S18 HPLC chromatogram of Oxali(Gem)(Au(I)) – Gen 2 - 0-100% acetonitrile in 5.84 min + 2 min constant 100% acetonitrile.

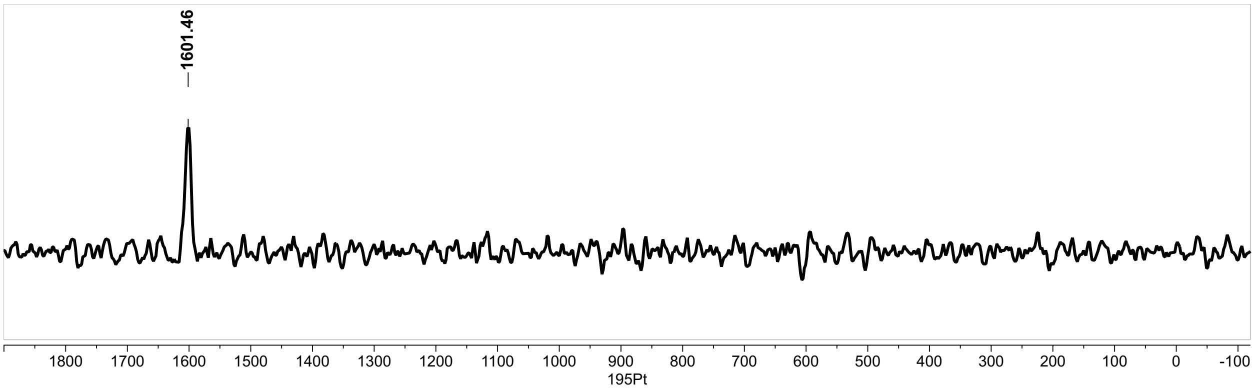

Figure S19 ^{195}Pt NMR spectrum of Oxali(Gem)(Au(I)) – Gen 2

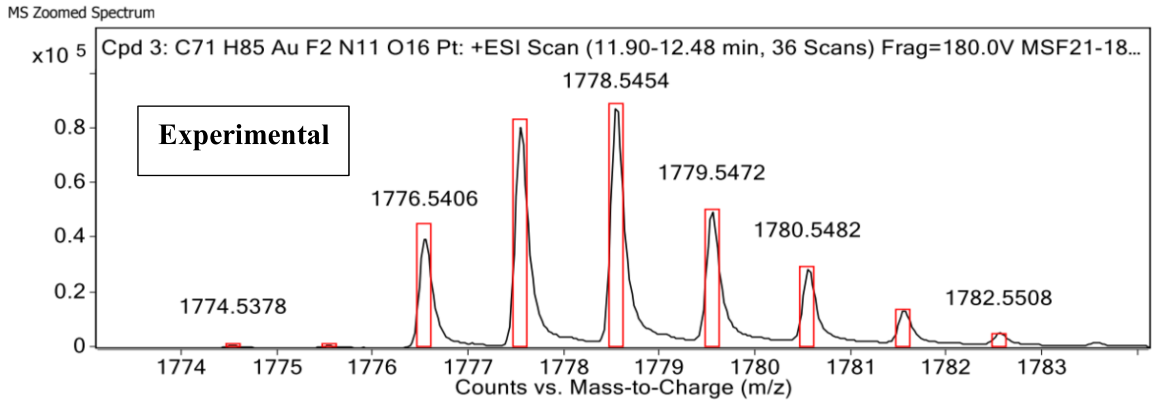

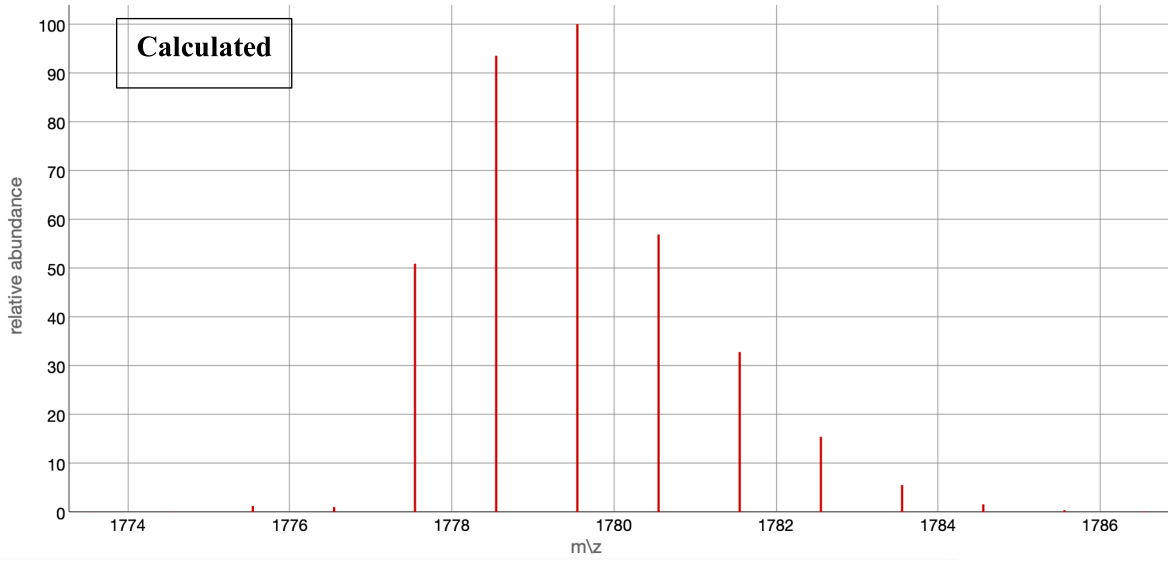

Figure S20 HRMS of Oxali(Gem)(Au(I)) – Gen 2

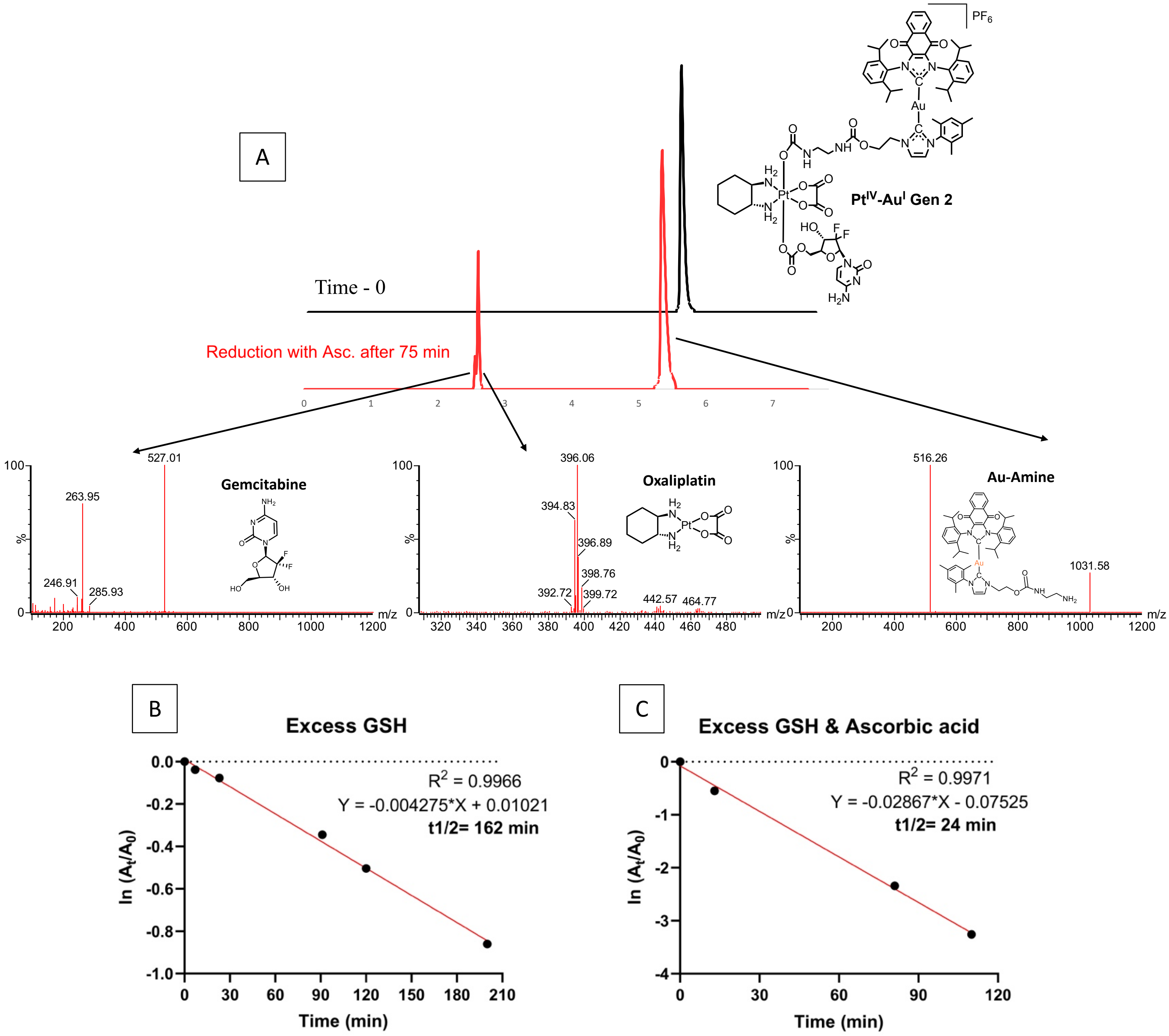

Figure S21 (A) Pt(IV)-Au(I) Gen 2 reduction products – Reduction of prodrug incubated for 75 min with 10 equiv. Asc. at pH 7 and 37 ºC as monitored via LC-MS. (B) Half-life of Pt(IV)-Au(I) Gen 2 in presence of excess GSH. (C) Half-life of Pt(IV)-Au(I) Gen 2 in presence of excess GSH and ascorbic acid.

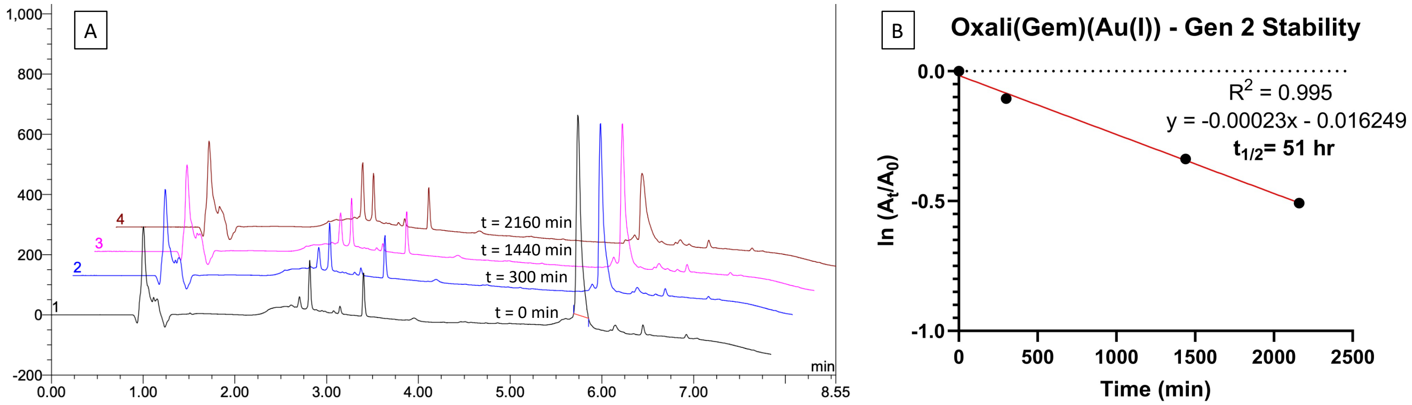

Figure S22 Stability study of Pt(IV)-Au(I) Gen 2 – A) HPLC chromatogram over time. B) Linear fitting of $\ln(A_t/A_0)$ vs time (t) for $t_{1/2}$ calculation.

Table S1 - IC_{50} values [μM] in A549 after a 72 h incubation period (tested in triplicate).

A549	IC_{50} (μM)
**Oxaliplatin**	0.87 ± 0.14
**Au(I)-Amine**	1.52 ± 0.17
**Gemcitabine**	0.006 ± 0.0007
**Pt-Au Gen 1**	1.01 ± 0.03
**Pt-Au Gen 2**	0.007 ± 0.001

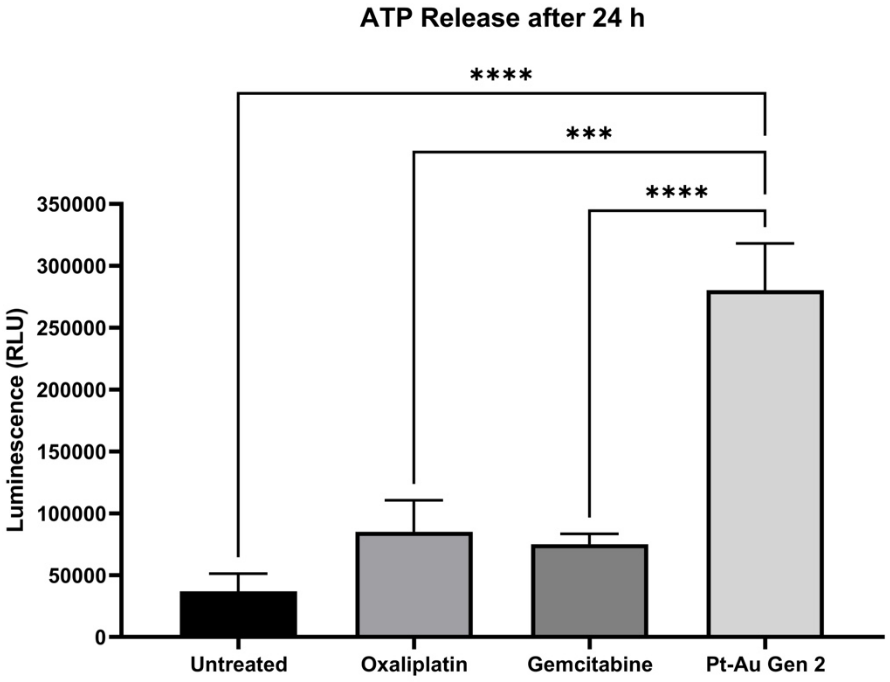

Figure S23 Extracelluar ATP release

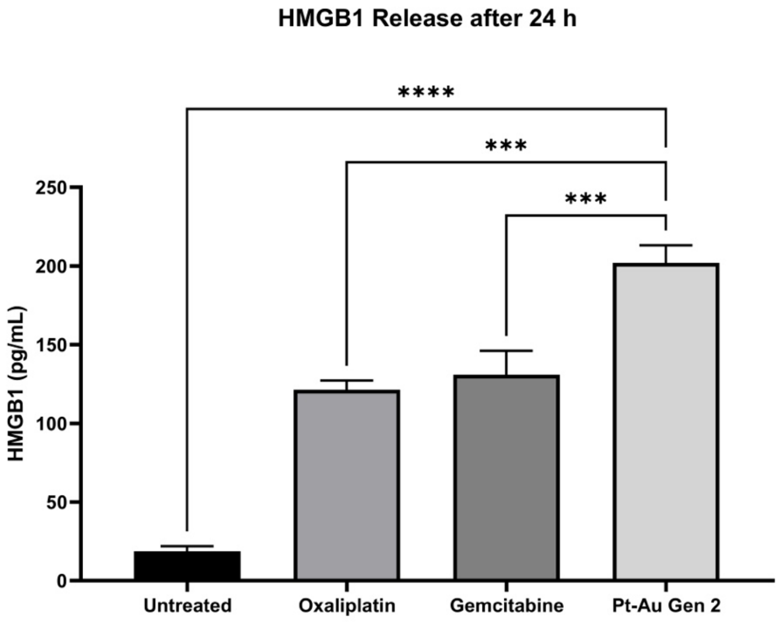

Figure S24 HMGB1 release

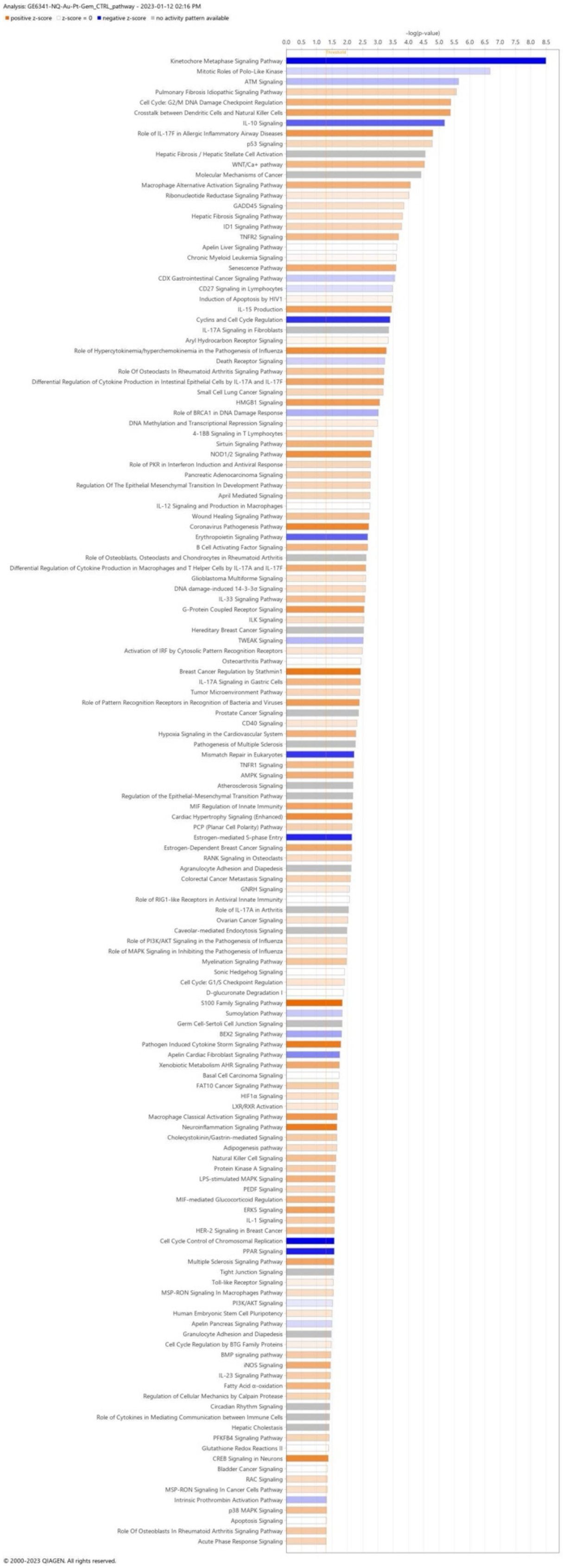

© 2000-2023 QIAGEN. All rights reserved.

Figure S25 RNA pathway analysis of Oxali(Gem)(Au(I)) – Gen 2 versus untreated

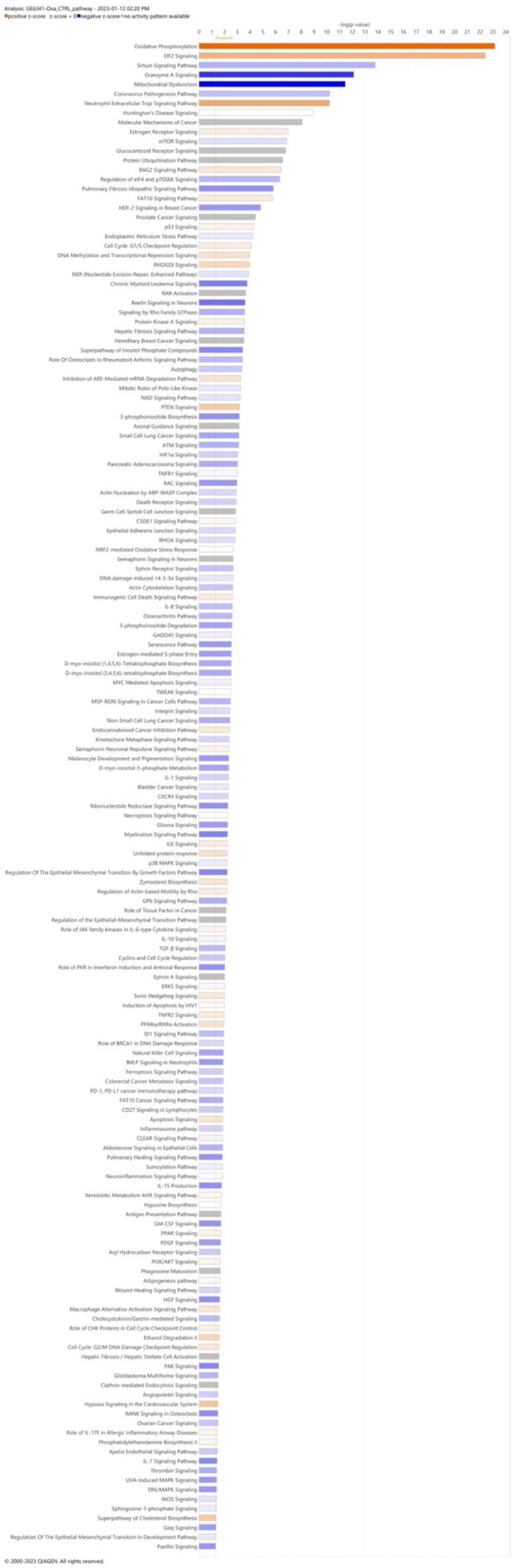

Figure S26 RNA pathway analysis of oxaliplatin versus untreated CT26 cells

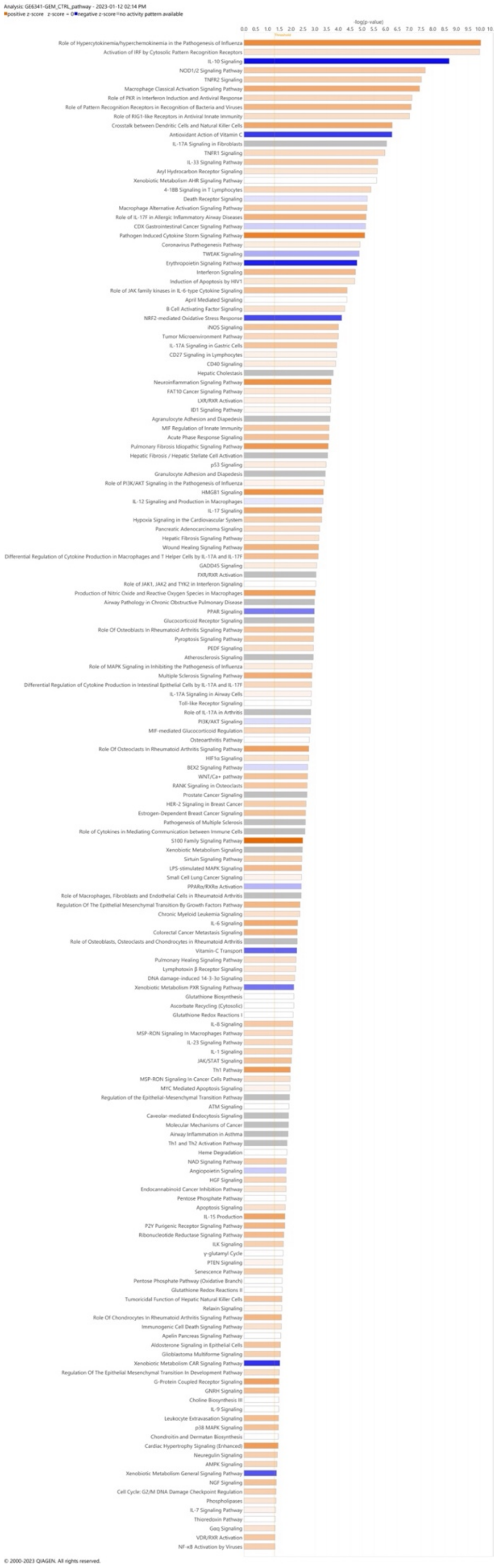

Figure S27 RNA Pathway Analysis of Gemcitabine versus untreated CT26 cells

Cell Cycle: G2/M DNA Damage Checkpoint Regulation	HMGB1 Signaling
AURKA	CSF2
BORA	FOS
CCNB1	IL11
CCNB2	IL15
CDC25B	IL17F
CDK1	IL1A
CDKN1A	IL6
CHEK1	JUN
CKS1B	LIF
GADD45A	MAP2K6
MDM2	PIK3R3
PLK1	PLAT
TOP2A	REL
WEE1	RELA
Crosstalk between Dendritic Cells and Natural Killer Cells	RELB
ACTA2	RHOD
ACTB	RHOJ
ACTG1	RND1
CAMK2A	RND2
CCR7	SELE
CD80	SERPINE1
CSF2	TGFB2
FAS	IL-10 Signaling
FSCN1	BHLHE40
HLA-A	CCND1
HLA-DRB5	CD80
IFNB1	CDKN1A
IL15	CDKN2D
IL6	CREB1
REL	CREB3L3
RELA	CREB5
RELB	DDIT4
p53 Signaling	DUSP1
APAF1	FOS
BBC3	HLA-A
BIRC5	HLA-DRB5
CCND1	IL1A
CCNG1	IL6
CDKN1A	INPP5D
CHEK1	JUN
FAS	MAP2K6
GADD45A	NFKBIA
GADD45B	NFKBIB
GADD45G	PKM
HDAC9	PRDM1
JUN	REL
MDM2	RELA
PIDD1	RELB
PIK3R3	Mitotic Roles of Polo-Like Kinase
THBS1	CCNB1
TNFRSF10A	CCNB2
TP53INP1	CDC20
TNFR2 Signaling	CDC25B
BIRC3	CDK1
FOS	ESPL1
JUN	FBXO5
NFKBIA	FZR1
NFKBIB	KIF11
REL	KIF23
RELA	PLK1
RELB	PLK2
TRAF1	PLK3
	PLK5
	PPP2R2C
	PRC1
	PTTG1
	WEE1

Figure S28 RNA Microarray Analysis –Associate Genes with Canonical Pathways

References

1. S. Sen, S. Hufnagel, E. Y. Maier, I. Aguilar, J. Selvakumar, J. E. DeVore, V. M. Lynch, K. Arumugam, Z. Cui, J. L. Sessler and J. F. Arambula, *J. Am. Chem. Soc.*, 2020, **142**, 20536–20541.
2. T. Babu, M. S. Levine, S. Acharya, E. Y. Maier and J. L. Sessler, *Angew. Chem. Int. Ed.*, e202514351.
3. T. Yempala, T. Babu, S. Karmakar, A. Nemirovski, M. Ishan, V. Gandin and D. Gibson, *Angew. Chem. Int. Ed.*, 2019, **58**, 18218–18223.
4. B. Dos Santos, M. C. Bion, M. Goujon-Svrzic, P. Maher and A. L. Dafre, *Anal. Biochem.*, 2024, **687**, 115445.
